# Cross talk between bacterial and human gene networks enriched using ncRNAs in IBD disease

**DOI:** 10.1038/s41598-023-34780-x

**Published:** 2023-05-11

**Authors:** Mohammad Elahimanesh, Mohammad Najafi

**Affiliations:** 1 Clinical Biochemistry Department, Faculty of Medical Sciences, Iran University of Medical Sciences, Tehran, Iran; 2 Microbial Biotechnology Research Center, Iran University of Medical Sciences, Tehran, Iran

**Keywords:** Microbiology, Molecular biology, Systems biology

## Abstract

Inflammatory bowel disease (IBD) is a long-term inflammatory immune-mediated gut illness with several extra-intestinal complications. The aims of this study were to identify a novel network-based meta-analysis approach on the basis of the combinations of the differentially expressed genes (DEGs) from microarray data, to enrich the functional modules from human protein–protein interaction (PPI) and gene ontology (GO) data, and to profile the ncRNAs on the genes involved in IBD. The gene expression profiles of GSE126124, GSE87473, GSE75214, and GSE95095 are obtained from the Gene Expression Omnibus (GEO) database based on the study criteria between 2017 and 2022. The DEGs were screened by the R software. DEGs were then used to examine gene ontology (GO) and the Kyoto Encyclopedia of Genes and Genomes (KEGG) pathways. The ncRNAs including the miRNAs and ceRNAs were predicted on the PPIs visualized using Cytoscape. Enrichment analysis of genes with differential expression (n = 342) using KEGG and GO showed that the signaling pathways related with staphylococcus aureus and pertussis bacterial infections may stimulate the immune system and exacerbate IBD via the interaction with human proteins including Fibrinogen gamma chain (FGG), Keratin 10 (KRT10), and Toll like receptor 4 (TLR4). By building a ceRNA network, lncRNA XIST and NEAT1 were determined by affecting common miRNAs, hsa-miR-6875-5p, hsa-miR-1908-5p, hsa-miR-186-5p, hsa-miR-6763-5p, hsa-miR-4436a, and hsa-miR-520a-5p. Additionally, the chromosome regions including NM_001039703 and NM_006267, which produce the most potent circRNAs play a significant role in the ceRNA network of IBD. Also, we predicted the siRNAs that would be most effective against the bacterial genes in staphylococcus aureus and pertussis infections. These findings suggested that three genes (FGG, KRT10, and TLR4), six miRNAs (hsa-miR-6875-5p, hsa-miR-1908-5p, hsa-miR-186-5p, hsa-miR-4436a, hsa-miR-520a-5p, and hsa-miR-6763-5p), two lncRNAs (XIST and NEAT1), and chromosomal regions including NM_001039703 and NM_006267 with the production of the most effective circRNAs are involved in the ncRNA-associated ceRNA network of IBD. These ncRNA profiles are related to the described gene functions and may play therapeutic targets in controlling inflammatory bowel disease.

## Introduction

Both Crohn disease (CD) and ulcerative colitis (UC), two primary types of inflammatory bowel disease (IBD), are defined by an abnormal immunological response of the intestinal mucosa. Current research on the development of diseases points to a complicated interplay between numerous environmental and genetic factors^1^. IBD is associated with periods of stomach pain, fever and fatigue, loss of appetite, bloody stool, weight loss, and a stimulation of immune system cells that cause inflammation by producing cytokines^2,3^. IBD is triggered by a combination of environmental variables, infectious pathogens, and genetic predisposition, which results in abnormal immune reactions to intestinal mucosa^4^. Inflammatory bowel disease has been on the rise over the past few decades, and since it can waste a society's human resources and render them ineffective, must be specified what causes it. This has enabled researchers to develop prevention strategies and therapeutic approaches^5^.

In the past, some studies investigated external factors affecting the incidence of inflammatory bowel disease, including the amount of smoking in the occurrence or exacerbation of symptoms in patients with inflammatory bowel disease^6,7^. According to particular research, altering the community's diet has affected the population of the intestinal microbiome, which has triggered the immune system to respond to this microbiome and to result in intestinal inflammation^8,9^. Polovsky's experiment showed that whereas lactobacillus and bifidobacterium levels reduce in CD patients compared with control subjects, bacteroidetes levels increase^2^. According to research, enterobacteria levels considerably rose in CD patients^10^. Several investigations indicated an association between staphylococcus aureus and pertussis infections and the development or worsening of symptoms in IBD patients^11–13^. It seems that more studies should be done in order to determine the molecular mechanism and exact path of inflammatory bowel disease.

On the other hand, numerous non-coding RNAs (ncRNAs), including long non-coding RNAs (lncRNAs), circular RNAs (circRNAs), and micro RNAs (miRNAs), have recently been linked to the etiology of inflammatory bowel disease (IBD)^14,15^. Involved in IBD pathobiology, ncRNAs are essential gene regulators at the transcriptional and translational stages^16^. The miRNAs have received the most attention out of all of ncRNAs, and IBD has been discovered to have many altered miRNA expression profiles^17^. The modulation of cytokines and chemokines with miRNAs, it is known that aberrant autophagy, intestinal epithelial permeability, and activation of the necrosis factor-B (NF-B) are suggested as other inflammatory mechanisms in IBD^18–20^. LncRNAs and circRNAs are knowing essential for gene regulations^21,22^. Several circRNAs and lncRNAs have also been discovered as biomarkers for tumor prediction and diagnosis^23,24^. It seems that investigating the profiles of noncoding RNAs such as circRNAs and lncRNAs on the gene sets can be an interesting and forward-looking field in the treatment of IBD. Moreover, recent research suggests that circRNAs and lncRNAs may be interesting therapeutic targets for a range of disorders^25^. The attention of IBD research will undoubtedly shift to lncRNA and circRNA.

Based on the above descriptions, the aim of study was to find the important modules on the gene enrichment using Gene Ontology (GO) and Kyoto Encyclopedia of Genes and Genomes (KEGG) studies in Gene Expression Omnibus (GEO) database. Then, to explore the critical miRNAs, lncRNAs and circRNAs on the gene networks. Additionally, we projected the effective siRNAs on the bacterial genes involved in the occurrence of IBD. This study was expected to reveal probable regulatory mechanisms behind the incidence of IBD.

## Materials and methods

### Microarray data

**Figure 1:**
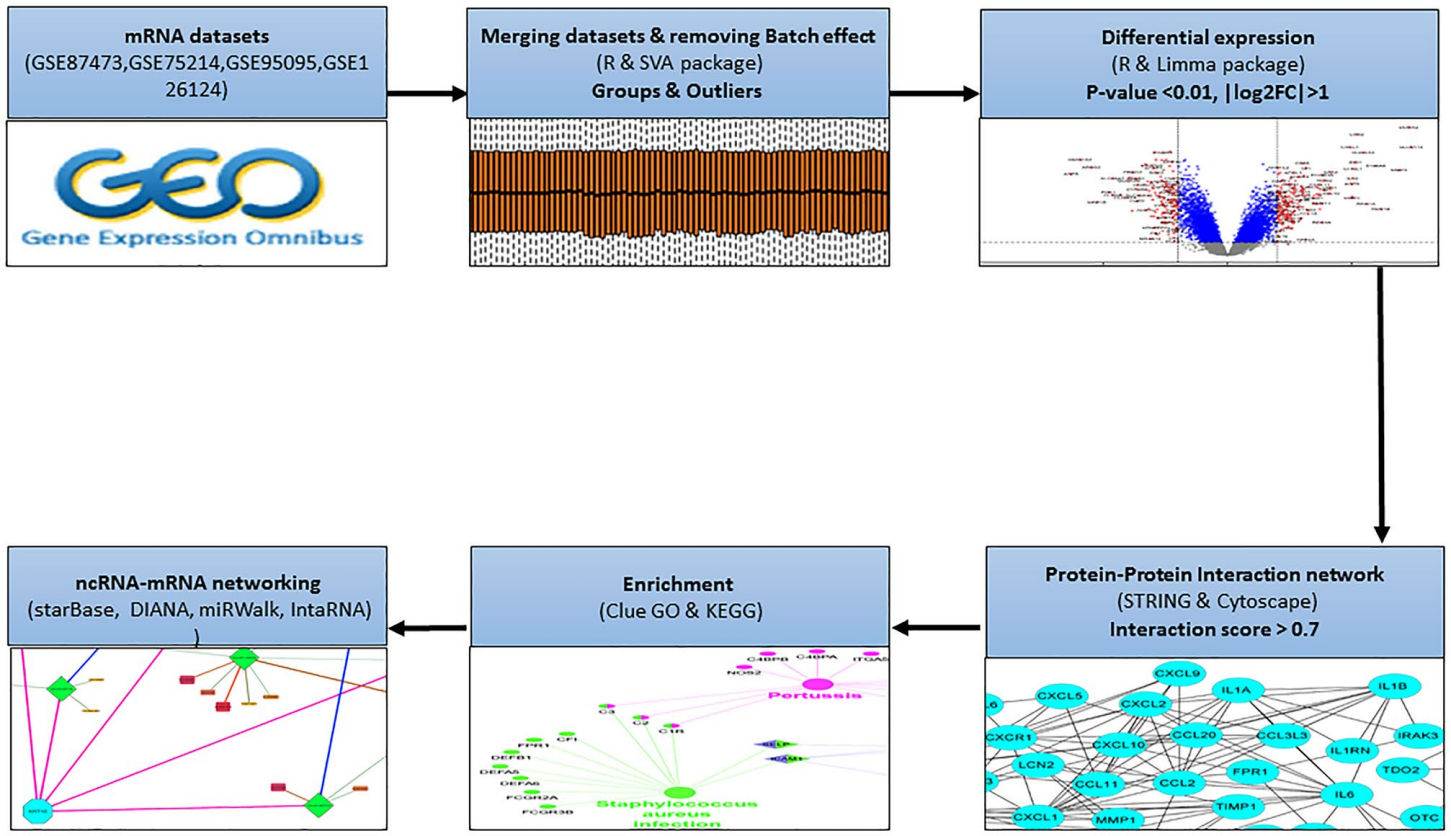
A flowchart representation of the steps involved in the dataset meta-analysis. The differentially expressed genes were identified based on four mRNA datasets. The protein–protein interaction network was generated using STRING database. The gene enrichment was done in Cytoscape software. The interaction between non-coding RNAs and hub genes was identified using databases of miRWalk, DIANA and starBase and finally, ceRNA networks were generated.

The following search terms were used to identify and download the mRNA microarray expression profile datasets from the GEO database (http://www.ncbi.nlm.nih.gov/geo) including inflammatory bowel disease, IBD, Homo sapiens (organism). The datasets were filtered on the data obtained from microarray technique, sample size > 50, gene size > 15,000 and without interventional agents between 2017 and 2022. The finalized microarray data for the meta-analysis were GSE126124 (GPL6244)^26^, GSE87473 (GPL13158)^27^, GSE75214 (GPL6244)^28^, and GSE95095 (GPL14951)^29^. Only samples from each dataset that met the following criteria were chosen to continue the analysis: (I) The GSMs had to be from colon and intestinal tissues (not blood). (II) The tissue should exhibit active inflammation (not inactive). (III) Without receiving any manipulations or treatments, samples from the IBD (Ulcerative colitis (UC) or Crohn's disease (CD)) group. Table 1 and Fig. 1 provided some details and descriptions of the used datasets and tools.

**Table 1:** Datasets for meta-analysis.

GEO	Control	IBD(UC/CD)	Experiment type	Platform	YEAR
GSE126124	21	57	Expression profiling by array	GPL6244	2019
GSE87473	21	106	Expression profiling by array	GPL13158	2018
GSE75214	22	133	Expression profiling by array	GPL6244	2017
GSE95095	36	24	Expression profiling by array	GPL14951	2019

### Protein–protein interaction (PPI) network

The Search Tool for the Retrieval of Interacting Genes (STRING; http://string-db.org) online database (version 11.5)^30^ was used to generate the PPI network, and the following parameters were used: a confidence score > 0.7 is applied as the threshold value to assess the interactions of protein pairs, databases, experiments, and text mining are the active interaction sources. Finding functional connections between proteins may shed light on the processes that give rise to disease development. Open-source bioinformatics software platform for visualizing molecular interaction networks is called Cytoscape (version 3.9.1) that was used to more analysis to find essential genes in the network based on the node and edge percentiles (75th) and network analysis with the Cytoscape tool "NetworkAnalyzer"^31^, which can calculate a comprehensive set of topological metrics for undirected and directed networks.

### Network functional enrichment

To identify significantly regulated functions of DEGs, the gene ontology (GO) enrichment analysis was carried out^32^. The systematic analysis, annotation, and visualization of gene functions were presented using the KEGG enrichment analysis. When P < 0.01 was statistically regarded as significant, GO enrichment and KEGG pathway analysis were performed using the Database for Annotation, Visualization, and Integrated Discovery (DAVID) (http://david.abcc.ncifcrf.gov) and the Cytoscape plug-in ClueGO^33^ to determine the biological significance of genes.

### ncRNA prediction

The gene hubs were found in the GO combination of the KEGG pathway crosses and other network features. The hubs included the genes connected between the body's signaling pathways and bacterial infections. MiRWalk (http://mirwalk.umm.uni-heidelberg.de), a comprehensive database of predicted and validated miRNA-target interactions, was used to predict relevant miRNAs that can modulate the hub genes (default parameters: species: human, Type of IDs: Gene Symbol, score = 1 (The score is calculated from a random-forest based approach by executing TarPmiR algorithm for miRNA target site prediction. Based on the training data, it's showing the probability that this interaction "works"), position = 3'UTR). The networks between the key genes and the most effective miRNAs were drawn on the specific criteria of each miRNA. In the next step, using the DIANA (https://diana.e-ce.uth.gr/home) (default parameters: Method: all, Validation type: direct, miRNA Conf. Level: High, lncRNA Source: RefSeq & Ensembl) and starbase database (https://starbase.sysu.edu.cn/index.php) (default parameters: clade: mammal, genome: human, assembly: hg19, Number of supporting Experiments$\ge$2), non-coding RNAs including circRNA and lncRNA, which play a role in regulating miRNA activity, were determined, and the interaction networks between these ncRNAs and miRNAs were created by Cytoscape software.

### siRNA prediction

The study looked in the proteins of staphylococcus aureus and bordetella pertussis that were interacted with human proteins to trigger or exacerbate the bacterial infections. The bacterial gene sequences involved in the cellular signaling transfection were scanned to predict the nucleotide positions with greater energetic stability for designing of the siRNA using IntaRNA database (http://rna.informatik.uni-freiburg.de/IntaRNA/Input.jsp) (default parameters: Number of interactions per RNA pair: 5, Suboptimal interaction overlap: can overlap in query, No GU at helix ends: Yes, No lonely base pairs: Yes, Min. number of base pairs in seed:7, Ignore seeds with GU ends: Yes).

### Statistical analysis and data-processing

The R software tool and the Affy package^34^ were used to normalize and standardize the raw data after they had been retrieved from the GEO database (Supplement 1). In expression analysis, normalization and data processing are crucial procedures since they could have an impact on the results of the meta-analysis. Each of the gene samples (GSM) underwent a log2 transformation. Then, the four datasets were merged and combat procedures from the SVA package^35^ were applied to the merged data to reduce study-specific batch effects (batch correction). Using the LIMMA package^36^, Student's t*-*test was done and genes with differential expression levels between the two groups of controls and IBD patients were identified. DEGs were defined as genes with a P-value < 0.01 and |log2FC|> 1.

## Results

### DEGs in IBD

**Figure 2:**
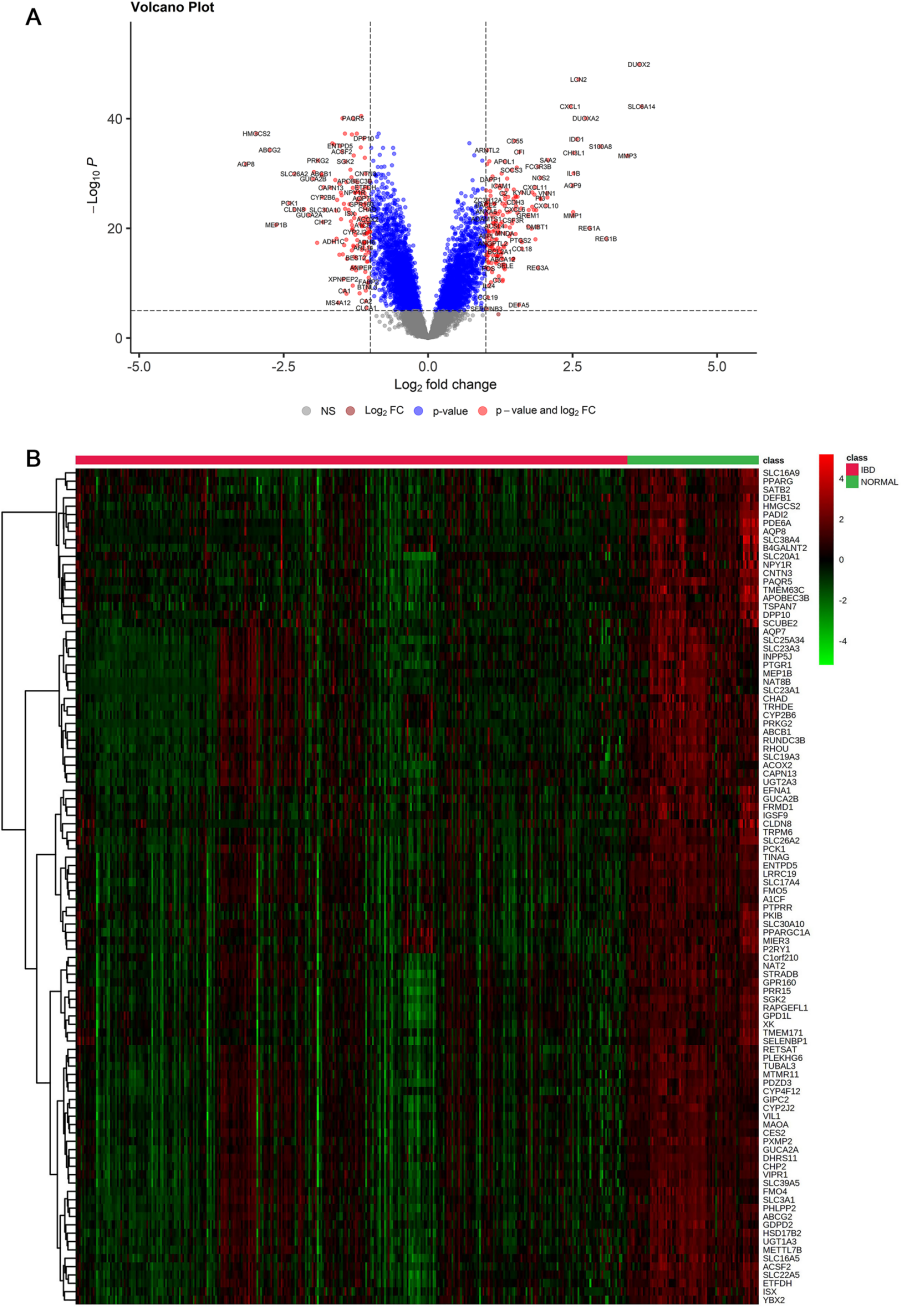

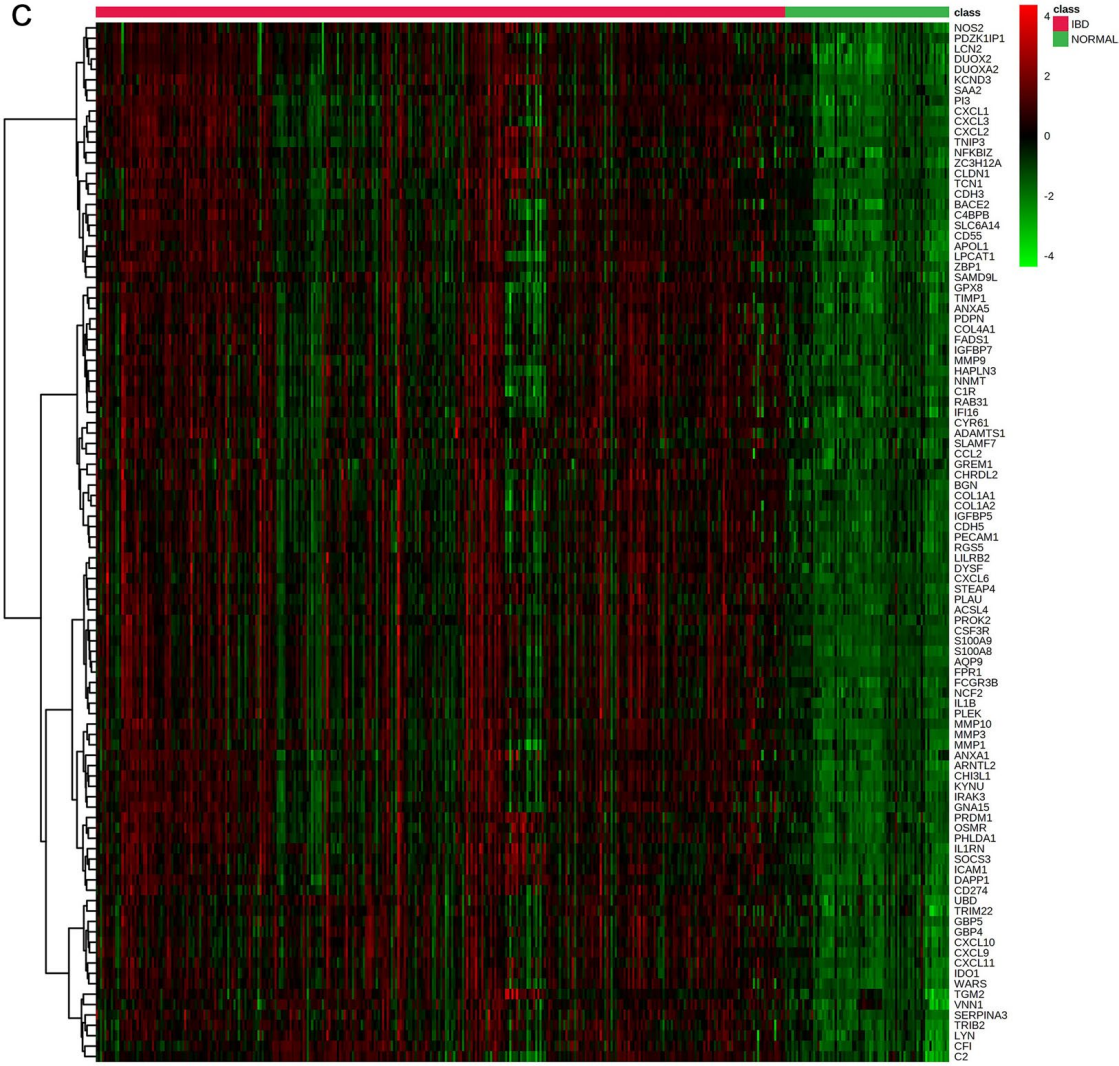
Different expression genes (DEGs) screening. (**A**) Genes which were differentially expressed (DEGs) between the control and IBD groups are displayed on volcano plots. The cut-off criterion was |log2 FC|> 1 and P < 0.01. The top 100 genes with decreased (**B**) and increased (**C**) expression levels in samples of IBD compared to control were shown in a heatmap. MetaboAnalyst 5.0 (https://www.metaboanalyst.ca/) server was used to generate heatmaps.

**Figure 3:**
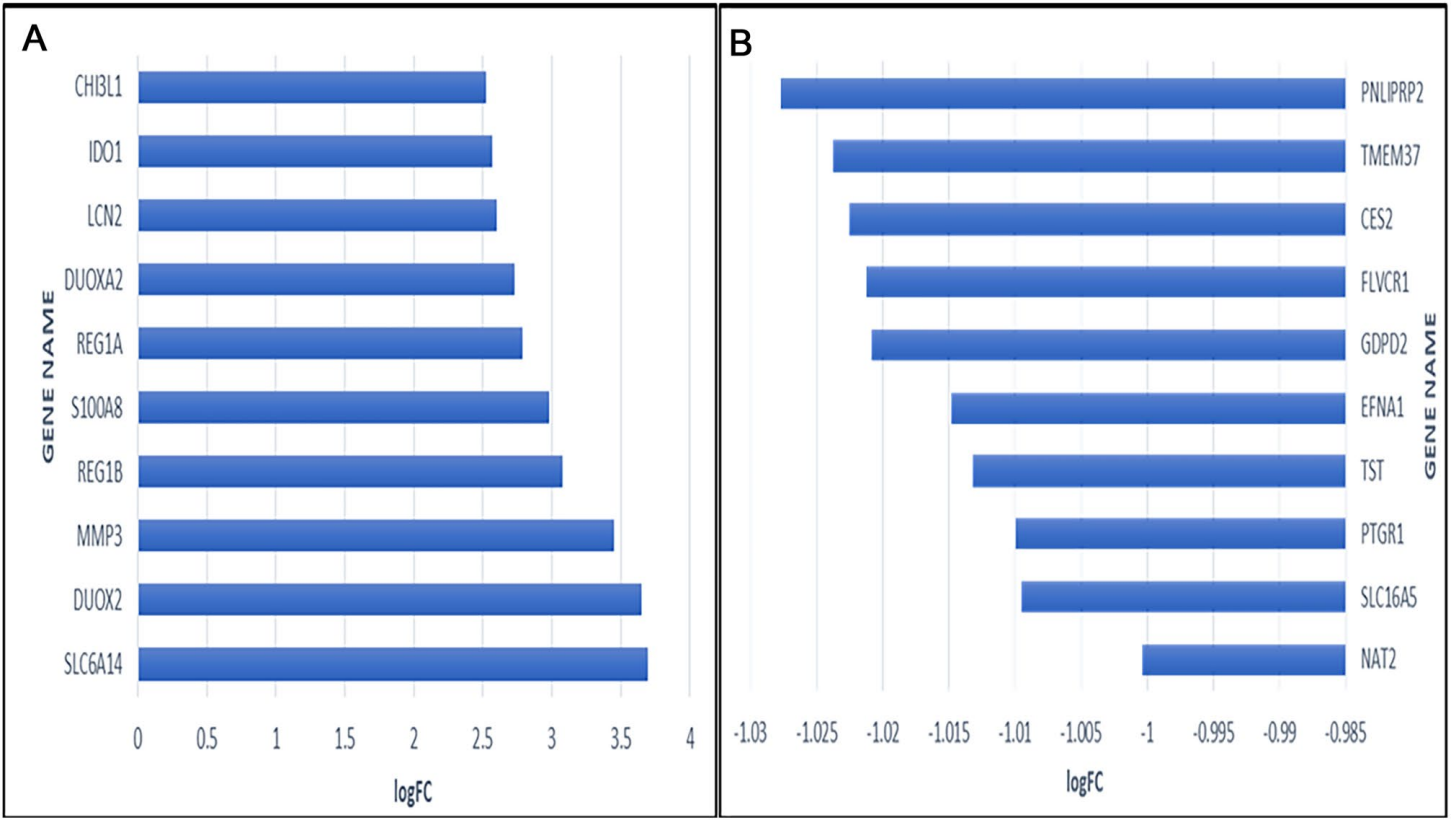
Top 10 up-regulated (**A**) and top 10 down-regulated genes (**B**) based on logFC values.

After downloading the GSEs (Table 1), and performing the steps of preprocessing, normalization, and merging of the datasets and removal of batch effects, a total of 396 samples were selected. Out of all the samples, 320 were related to people with inflammatory bowel disease and 76 samples were related to healthy people. A total of 342 DEGs were found comprising 149 downregulated genes and 193 upregulated genes in the IBD vs. control samples (Figs. 2 and 3).

### Crucial genes were created on the DEG network

**Figure 4:**
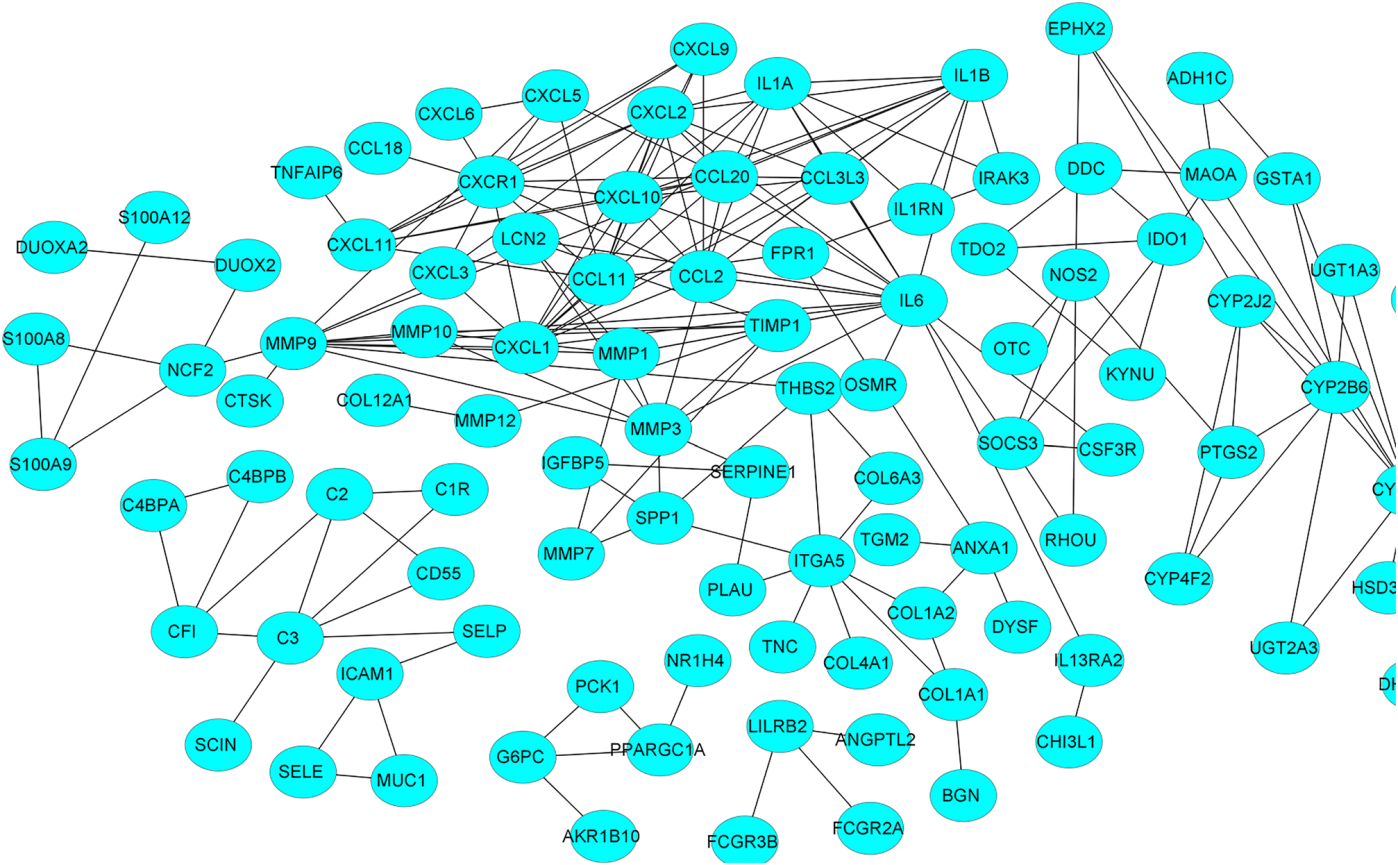
The top 75% module of the DEG network of protein–protein interactions for IBD. The nodes and edges were considered with confidence scores and logFC above the third percentile. The network consisted of 118 nodes and 211 edges. An enrichment analysis was performed on the genes in the network.

A primary network of DEGs (differentially expressed genes) was created using active interaction sources such as experiments, text mining, databases, and interaction scores greater than 0.7 from the STRING server. Then, the network was visualized in Cytoscape software (Supplement 2, nodes 188, edges 403). Additional studies were conducted to discover more crucial genes in the network (118 nodes and 211 edges) on the percentiles (75th) of the node (logFC of each gene) and edge (the sum of the scores of the edges in STRING server) scores (Fig. 4). The sub-networks containing 3 or less than 3 nodes were removed, and the final network were used for enrichment analysis.

### DEG subnetworks were enriched using GO and signaling pathways

**Figure 5:**
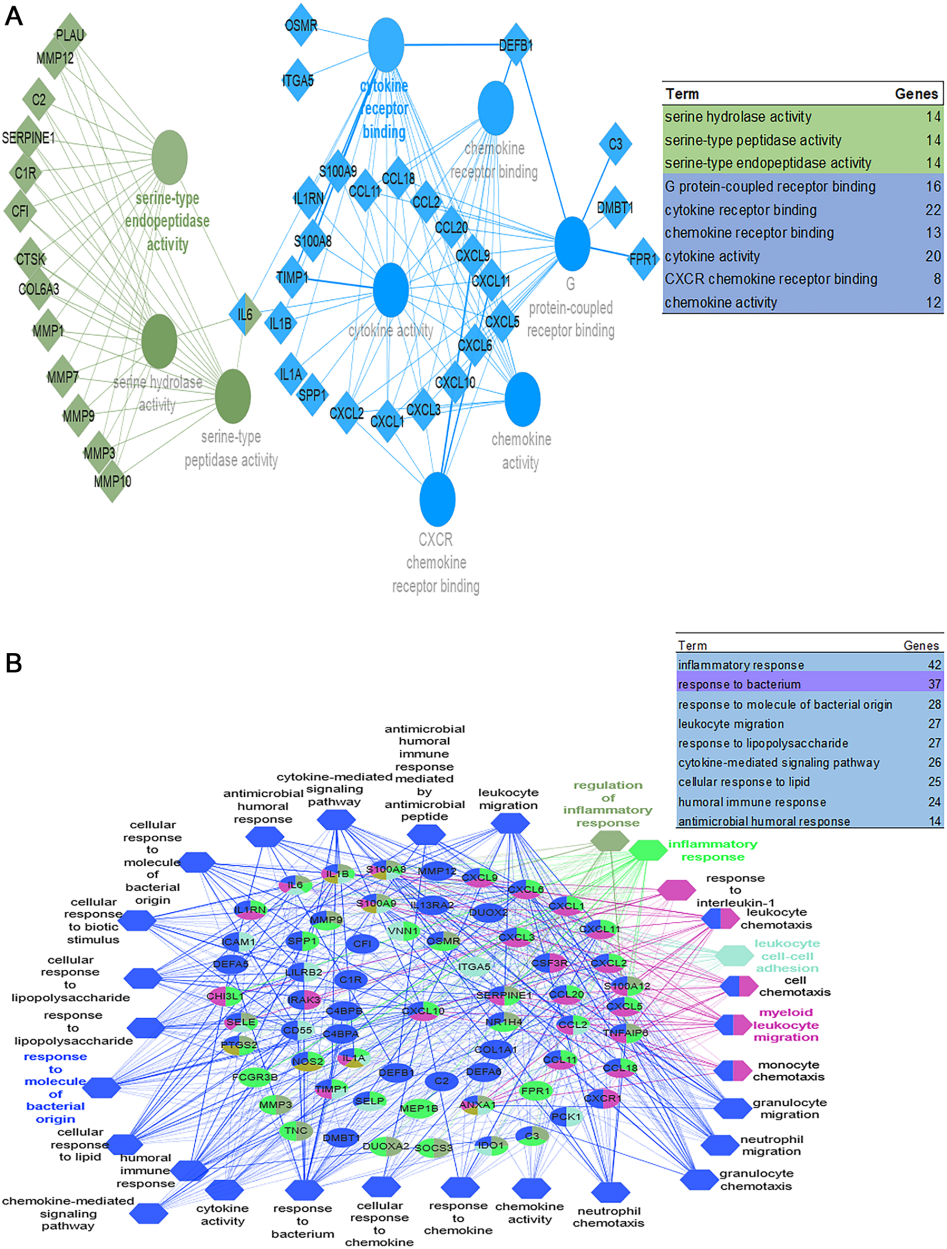
Gene Ontology enrichment. (**A**) Molecular function. Most of the genes in the network are located in cytokine receptor binding (22 genes), cytokine activity (20 genes) and G protein-coupled receptor binding (16 genes). (**B**) Biological process. In terms of biological process, a large number of genes are present in response to bacteria (37 genes), inflammatory response (42 genes), and response to molecules of bacterial origin (28 genes).

**Figure 6:**
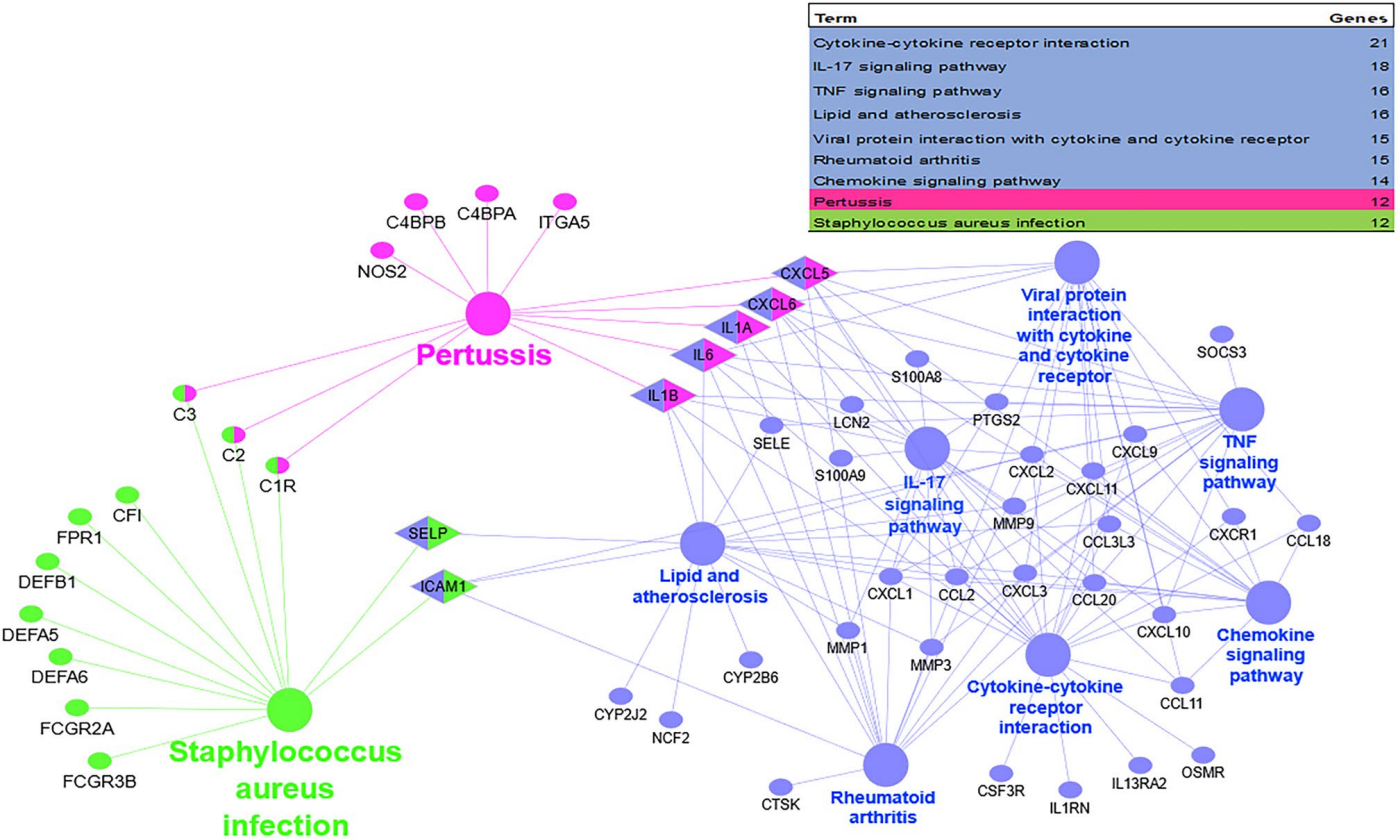
KEGG-based enrichment and identification of biological pathways. Genes are generally located in 3 separate pathways including the set of paths leading to the response of the immune system and inflammation of the human body (blue node and edges), and the path of bacterial infections by bordetella pertussis (pink node and edges) and staphylococcus aureus (green node and edges).

Using the ClueGO and DAVID tools, functional annotation and pathway analyses, including GO (Biological Process, Molecular Function) and KEGG analyses, were carried out to further perform a systematic characterization and investigate the biological functions of the identified DEGs in IBD vs. control samples (Fig. 5, supplement 3). According to the findings of the gene ontology analysis, the genes with significant differences were enriched in the extracellular matrix structure, regulation of complement activation, chronic inflammatory response, regulation of apoptotic cells, inflammatory response, and response to vitamin. The DEGs in IBD were then found to be primarily enriched in pertussis, staphylococcus aureus infection, cytokine-cytokine receptor interaction, IL-17 signaling, and viral protein interaction with cytokine and cytokine receptor, according to KEGG pathway analysis (Fig. 6).

### Associations of bacterial and human proteins were determined

**Figure 7:**
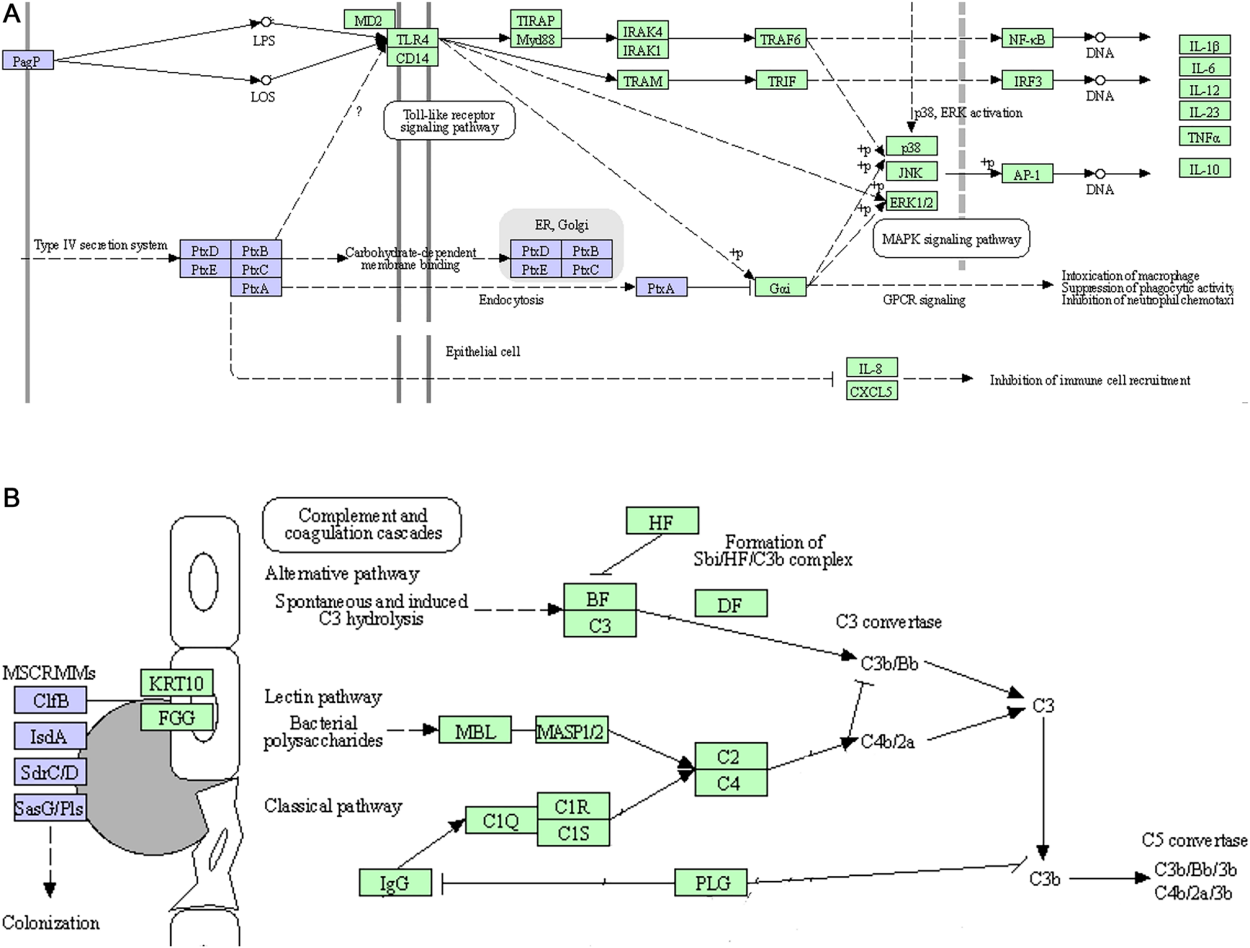
The KEGG upstream pathways in inflammatory bowel disease. (**A**) Pertussis bacterial genes ptxA, ptxB, ptxC, ptxD, ptxE, and pagP (purple) can interact with human genes MD 2, CD 14, and TLR4 (green) to cause infection and inflammation (hsa05133). (**B**) ClfB, IsdA, SdrC, SdrD, and SasG (purple) are bacterial genes of staphylococcus aureus that can interact with human genes FGG and KRT10 (green) (hsa05150).

By considering KEGG enrichment (Fig. 6) and examining the pathways of bacterial infections in humans, it was found that bordetella pertussis infection by association with human proteins (CXCL5, CXCL6, IL6, IL1B, IL1A) and staphylococcus aureus infection by association with 2 human proteins (ICAM1, SELP) affect inflammatory reactions in the human host (Fig. 7). Regarding the upstream axes of SELP and ICAM proteins, it was discovered that the infection is started by bacterial proteins (ClfB, IsdA, sdrC, sdrD, and SasG) that are interacted to human proteins (KRT10, FGG), and actually results in an infection that stimulates the inflammatory systems in the human (Fig. 7A). Additionally, the human upstream proteins of CXCL5, CXCL6, IL6, IL1B, and IL1A were evaluated in the process of pertussis infection. The bacterial PtxE, PagP, PtxD, PtxC, PtxA, and PtxB proteins are crucial by interacting with the human proteins MD2, CD14, and TLR4. These key proteins promote the development and spread of infections (Fig. 7B).

### ncRNAs were predicted for human genes

**Figure 8:**
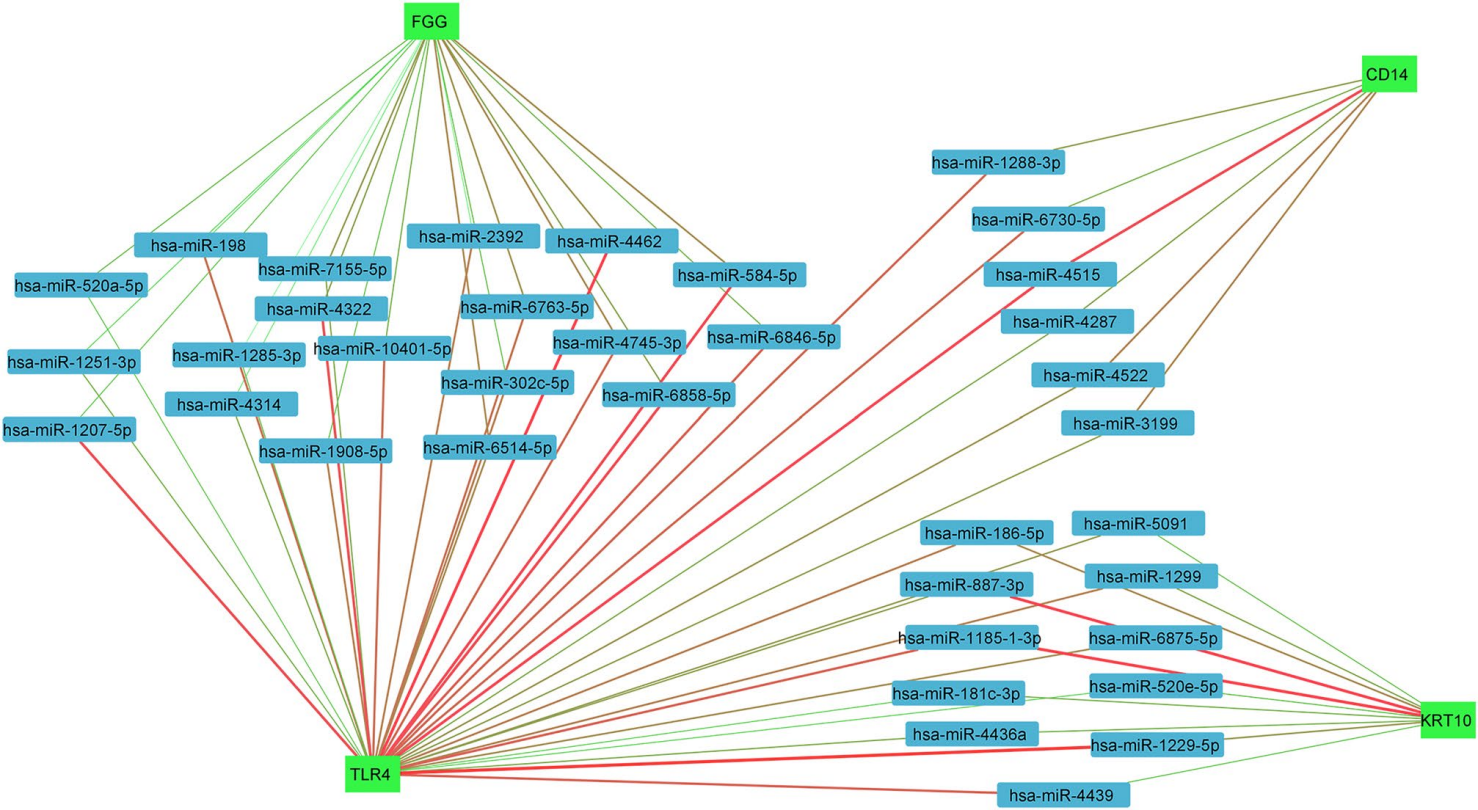
The network of miRNAs and human genes involved in the staphylococcus aureus and bordetella pertussis bacterial infections. The pertussis and staphylococcus aureus pathways transduced the message via CD14, TLR4, KRT10 and FGG. The edge (from green to red) showed a rise in the trustworthiness of the connections between nodes. The important miRNAs affecting genes KRT10 and TLR4 predicted hsa-miR-1229-5p, has-miR-887-3p and hsa-miR-1185-1-3p. The important miRNAs targeting of genes of CD14 and TLR4 predicted hsa-miR-4515. However, the important miRNAs affecting genes FGG and TLR4 suggested hsa-miR-4462, hsa-miR-584-5p and has-miR-1207-5p.

**Figure 9:**
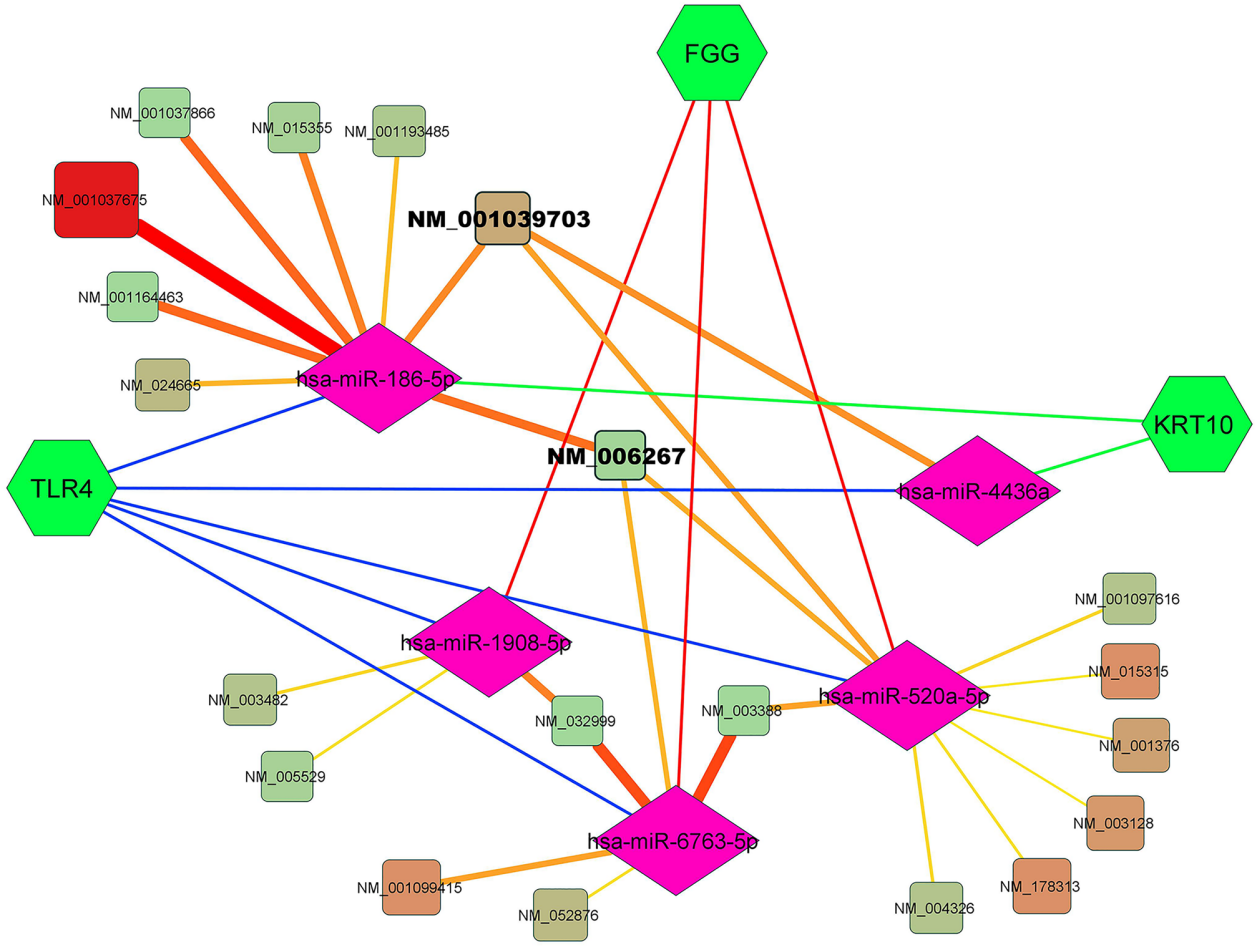
Communication network between human genes involved in bacterial infections (green nodes), miRNAs (pink nodes) and circRNAs. The edges between miRNAs and circRNAs (yellow to red) increased on the numbers of circRNAs corresponding to that chromosomal region that affects the miRNAs. The circRNAs nodes (from green to red) are also based on the experimental and laboratory information of the presence of circRNAs in that chromosomal region. The chromosomal regions including NM_001039703 and NM_006267 had the greatest effects on the interaction network between miRNAs and human genes involved in staphylococcus aureus and pertussis bacterial infections.

Small noncoding RNAs called microRNAs (miRNAs) were chosen for FGG, KRT10, MD2, TLR4, CD14 genes in the pathway upstream. In miRNA-gene network, the edges were weighted by defining of sum of the scores (accessibility, me, and energy) of each miRNA in the miRWalk database (Supplement 4). The network was finally limited on the miRNAs that were shared among genes (Fig. 8). The lncRNAs were determined on the network of interactions between common miRNAs and genes (Supplement 5). In addition, the interactional network was created between common miRNAs and the most effective circRNAs that regulate their activity (Fig. 9).

### siRNAs were predicted for bacterial genes

**Figure 10:**
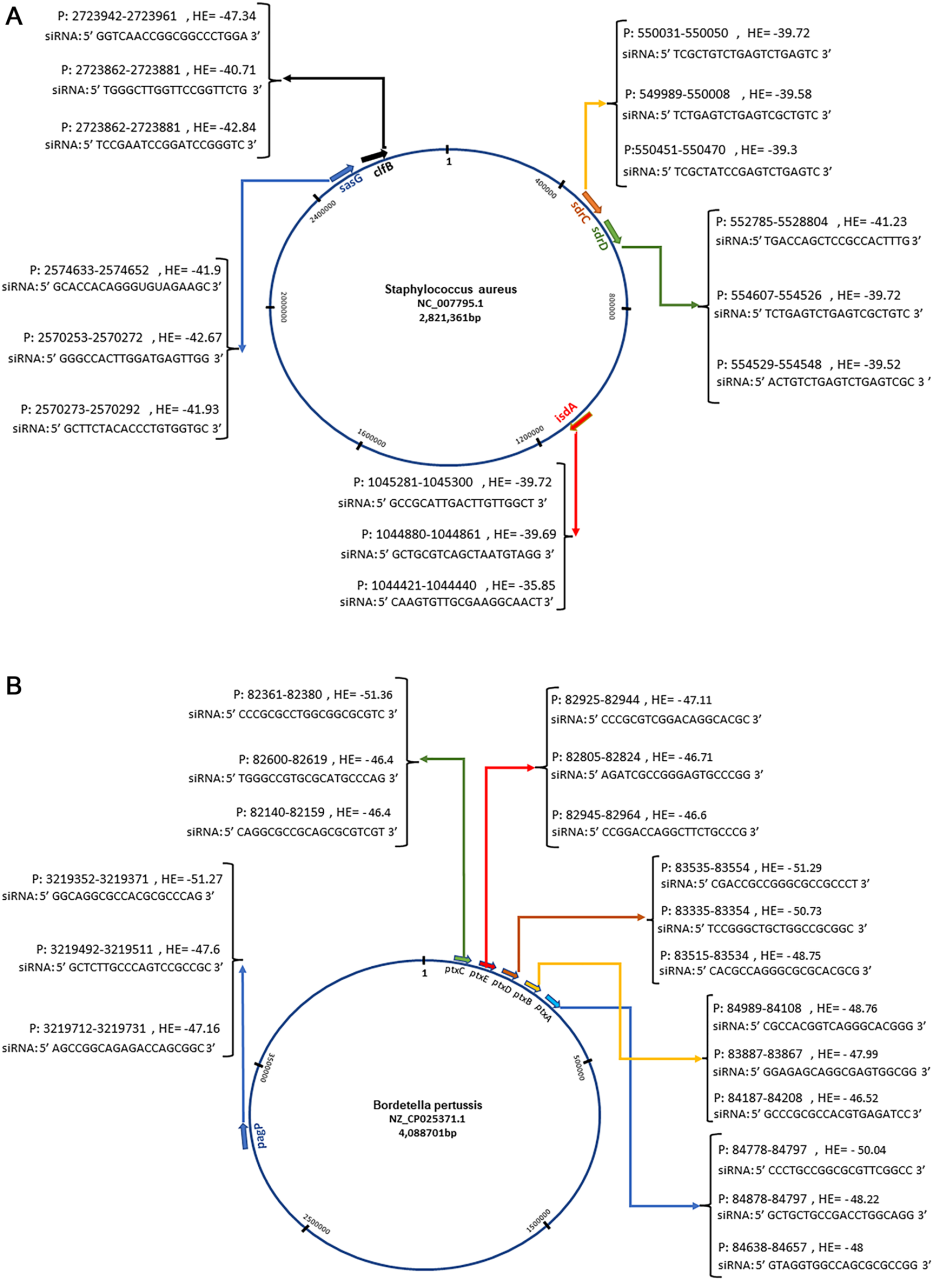
The potential chromosomal sites for designing of antisense RNA. Staphylococcus aureus (**A**) and Bordetella pertussis (**B**). The results displayed the top three positions on each gene based on the hybridization energy by the interaction between siRNA. P, position; EH, Hybridization Energy (Kcal/mol).

The bacterial proteins are proposed in staphylococcus aureus (ClfB, IsdA, sdrC, sdrD, and SasG), and in bordetella pertussis (PtxE, PagP, PtxD, PtxC, PtxA, and PtxB) in IBD. The complete genomes of staphylococcus aureus and bordetella pertussis were taken from the NCBI database. The nucleotide sequence of the genes (ClfB, IsdA, sdrC, sdrD, and SasG, PtxE, PagP, PtxD, PtxC, PtxA, and PtxB) involved in the occurrences of these infections in humans that may stimulate the path leading to inflammatory bowel disease were scanned for prediction of best positions for designing of siRNAs. In the intaRNA database, the most effective siRNAs on gene function were predicted based on the energy released from the hybridization of siRNA and mRNA (Fig. 10).

## Discussion

The types of inflammatory bowel disease (IBD) including both Crohn's disease (CD) and ulcerative colitis (UC), are characterized by an abnormal immunological response^37^. The development of IBD points to a complicated subject between numerous important factors^38^. The correct understanding of relationships between these factors clears the pathophysiology of IBDs. IBDs may therefore have potential diagnostic and therapeutic targets if the molecular mechanisms of IBDs are understood using high-throughput technologies, which has been widely employed to study the pathogenic processes of IBD^39,40^.

In the current investigation, 342 DEGs, including 193 upregulated and 149 downregulated genes, were identified in the IBD samples. The top 75% module of the network's DEGs was subjected to a GO analysis, and the results showed that DEGs were particularly enriched in cytokine activity, cytokine receptor binding, inflammation, response to bacteria, response to molecules of bacterial origin, leukocyte migration, response to lipopolysaccharide, and cytokine-mediated signaling pathways. The aforementioned GO keywords have been shown in prior research to be potentially significant events in the etiology of IBDs^27,39,41,42^.

According to the results of the KEGG pathway analysis, the DEGs were particularly enriched in the signaling pathways for cytokine-cytokine receptor interaction, IL-17, TNF, viral protein interaction, rheumatoid arthritis, lipid and atherosclerosis, chemokine, pertussis, and staphylococcus aureus infection. Previous studies have shown that the development of inflammatory bowel disease is caused by the interaction of immune system molecules (cytokines and interleukins, etc.), cells (macrophages, lymphocytes (B cells and T cells), etc.), signaling pathways (TNF signaling pathway and chemokine signaling pathway, etc.) and other factors (foreign microbes or immune system-stimulating drugs)^43,44^. Immunological system activation causes immune reactions, gastrointestinal system inflammation, and the onset of IBD symptoms^45^.

According to several studies, mycobacterium paratuberculosis stimulates the immune system's signaling pathways, which may trigger or aggravate inflammatory bowel disease^46–49^. Some studies have shown that by changing people's diets towards fast foods with high sugar, the microbial population of the digestive tract changed and harmful bacterial species (B. vulgatus, B. thetaiotamicron, E. coli invasive, Intestinal Helicobacter) replaced the beneficial bacterial species (Lactobacillus, Nonpathogenic E. coli, and S. boulardii) in the digestive tract, which stimulated the immune system and created a response to this harmful microbial population. This results in inflammation and gastrointestinal diseases like IBD^50,51^.

Although previous studies have found traces of bacterial infection by staphylococcus aureus and pertussis in IBD patients but the pathways and genes involved in the potential incidence of bowel inflammation by infectious agents of staphylococcus aureus and pertussis are interested to find^11–13,39^. About 50% of healthy adults have persistent or sporadic staphylococcus aureus colonization of the skin around their noses, but it has recently been identified as a key early colonizer of an infant's gut^52,53^. There is little information on the intestinal colonization and fecal transport of methicillin-resistant s. aureus (MRSA) in IBDs^54,55^.

In this study, the onset of inflammatory bowel disease due to the possibility that infections caused by staphylococcus aureus and pertussis were evaluated. According to the enrichment, there were identified the most susceptible pathways and genes that could be studied to prevent inflammation caused by the bacteria.

According to research, KRT10 (keratin type I cytoskeletal protein 10) increases in people with inflammatory bowel disease and physically alters and rearranges the structure of the gastrointestinal tract's epithelial cells. This alteration affects the permeability of the epithelial layer and the cell’s ability to self-renew. These conditions make it possible for pathogens to enter the body's interior layers and induce bacterial infection like IBD^56,57^. Some studies suggested that FGG (fibrinogen gamma chain) plays as an early mediator in the cross-talk between coagulation and inflammation, and it can stimulate immune system reactions by interacting with bacterial agents and immune system cells^58^.

The ability of staphylococcus aureus to adhesion to squamous epithelial cells was consistently shown to be influenced by ClfB (clumping factor B), IsdA (iron-regulated surface determinant protein A), SdrC (serine-aspartate repeat-containing protein C), and SdrD (serine-aspartate repeat-containing protein D). A mutant with no copies of all four proteins exhibited total adhesion defects. A vaccine against two or more of these surface proteins could significantly reduce carriage as well as the likelihood of infection and spread^59–62^. It has been demonstrated that staphylococcus aureus surface proteins SasG (surface protein G) and Pls (surface protein of certain methicillin-resistant Staphylococcus aureus strains), which were previously discovered through in vitro studies of genome sequences, also contribute to bacterial adhesion to epithelial cells, and in their presence, the adhesion strength of staphylococcus aureus to epithelial cells increases^63^.

The pertussis lipopolysaccharides (LPS) and lipooligosaccharides (LOS) are ligands for the receptor complexes that include the Toll-like receptor 4 (TLR4), MD-2, and CD14. Inflammatory and immunological defense responses are triggered by the stimulations of these receptor complexes, which activate signaling pathways like NF-KB and causes the expression of antimicrobial genes and the release of cytokines, which can lead to inflammation^64^. MD-2 is a small secreted glycoprotein that binds to the hydrophobic region of LPS and the extracellular domain of TLR4. TLR4 is activated by the interaction between MD-2 and LPS^65^. After LPS stimulation, CD14 inhibits the rise in barrier permeability and leads to activation of the NF-kB signaling pathway^66^.

A typical A-B toxin is pertussis toxin (PTX). ADP-ribosyl transferase activity is seen in the A-subunit (PTXA or S1 subunit). The B-subunit, which is made up of the S2–S5 subunits (PTXB, PTXC, PTXD, and PTXE), binds cell surface molecules to enable the toxin to enter the cells. The PTXA ADP-ribosylates the α subunits of heterotrimeric Gi/o proteins, preventing the Gαi/o proteins from inhibiting adenylyl cyclase (AC). One of the ways that PTX causes the many pathogenic consequences in host cells is by the alteration of the Gi/o proteins, which leads to an increased buildup of cAMP. An intracellular signal transduction cascade is activated when the B-subunit interacts with the Toll-like receptor 4^67,68^. A palmitate residue is transferred by PagP (lipid IVA palmitoyl transferase) from a phospholipid's sn-1 position to the N-linked hydroxymyristate on the proximal unit of lipid A. One outer membrane enzyme involved in the production of lipid A is PagP^69^.

Non-coding RNAs, including miRNAs, circRNAs, and lncRNAs, have been shown to play intricate roles in the development of IBD^15^. By binding to miRNAs in a competitive manner, circRNAs and lncRNAs can control the expression of genes. More and more research points the roles for circRNA-miRNA-mRNA axis or lncRNA-miRNA-mRNA axis in the development of disorder^70–72^. For example, by increasing the expression of miR-146b and its effect on the Siah2 gene, NF-KB pathway becomes more active and causes an increase in the intestine inflammation^73^. The increase of miR-126 also has the same effect by affecting the IκBα gene^19^. CircRNA_103516 in PBMCs is reported as an excellent potential biomarker for the diagnosis of IBD. Through hsa-miR-19b-1-5p sponging, dysregulation of circRNA_103516 may contribute to the molecular basis of IBD^74^. The discovery of lncRNA IFNG-AS1 as a new regulator of IFNG inflammatory responses and the deregulation of lncRNA signatures in UC suggest the potential significance of noncoding RNA pathways in the control of inflammatory bowel disease^75^.

In this study, five important human genes (KRT10, FGG, TLR4, CD14, and MD2) reported in the development and spread of pertussis and staphylococcus aureus infections. Using the miRWalk database, we created an interaction network between some genes (KRT10, FGG, TLR4) and common miRNAs. In addition, the results showed that NM_001039703 regions with miR-186-5p, miR-4436a, and miR-520a-5p and also NM_006267 regions with miR-186-5p, miR-6763-5p, and miR-520a-5p are hotspots that produce circular RNAs on the function of human genes effective in causing staphylococcus aureus and pertussis bacterial infections. Future experimental studies can clarify the effects of these circRNAs on the regulation of the function of these miRNAs and genes.

The ceRNA network including miRNA-lncRNA-mRNA showed that lncRNA XIST and NEAT1 have the most important role among other lncRNAs in the network by affecting five common miRNAs related to human genes involved in staphylococcus aureus infection and pertussis. Some studies investigated the effects of lncRNA XIST on immune response. Through modifying the expression of ASF1A and BRWD1, lncRNA XIST can act as a ceRNA to sponge hsa-miR-212-3p to control inflammation and apoptosis in the development of AKI (acute kidney injury)^70^. The expression of proinflammatory cytokines generated by E. coli or S. aureus was considerably boosted by XIST silencing. Additionally, NF-κB phosphorylation caused by E. coli or S. aureus and the formation of NLRP3 inflammasome were suppressed by XIST. However, there were some controversies on XIST function but these findings implied that XIST inhibits NF-κB pathway activation triggered by E. coli or S. aureus^76^. Another study suggested that the stimulatory action of XIST was related to NF-κB. In conclusion, OSAHS (obstructive sleep apnea/hypopnea syndrome) patients had inflammation in their adenoids that was mediated by the XIST-GRα-NF-κB signaling pathway^77^.

The lncRNA NEAT1 is associated to the innate immune response. It seems that this lncRNA has a function in triggering the immune response by activating the kappa pathway^15^. NEAT1 was highly expressed in IBD and played a role in the inflammatory response by controlling the intestinal epithelial permeability and polar macrophages via exosomes^78^. According to some researches, TNFRSF1B may contribute to colitis by promoting inflammation. It was discovered that NEAT1 plays a pro-inflammatory effect via controlling TNFRSF1B (tumor necrosis factor superfamily member 1B), and facilitates NF-κB p65 entrance into the nucleus^79^. Studies suggested that the Neat1-miRNA204-5p axis plays an important role in the regulation of the PI3K-AKT pathway, which plays an important role in activating the pathways that lead to more activity of the immune system^80^. Studies have shown that the increase of NEAT1 has been observed in various cancers, liver diseases, non-alcoholic fatty liver disease (NAFLD), and liver fibrosis^81^. Although most studies show an increase in NEAT in IBD, more experimental tests are needed to determine the exact role of lncRNA NEAT1. Other predicted lncRNAs (SNHG3, SNHG7, NORAD, AC023509.1, AC016876.2 and AC021078.1) can also be good subjects for further experimental tests to determine their exact role in bacterial infection and inflammation.

Considering that inhibiting bacterial genes in staphylococcus aureus and pertussis bacterial infections can prevent the infection process. siRNAs as silencing of these genes may be considered effective to deal with staphylococcus aureus and pertussis infections, there were able to identify the most stable and sensitive spots for the action of siRNAs. The siRNA was functional in lowering the bacterial burden in a mouse model of hematogenous lung infection, according to the in vivo results. The use of siRNA to target coagulase looked to be a novel method of treating MRSA (Methicillin-resistant Staphylococcus aureus) infections^82^. It was shown that rectal administration of siRNA targeting TNF-a resulted in relative mucosal resistance to experimental colitis using a mouse model of inflammatory bowel disease^83^. Nanoparticles with surface CD98 antibody and CD98 siRNA (siCD98) loaded on them suppressed the production of this protein in colonic epithelial cells and macrophages^84^.

In conclusion, the gene hubs obtained from cross talking of human and bacterial pathways were the subjects to find the ncRNA profiles. These regulatory sets can simultaneously affect the human and bacterial gene groups and may be effective therapeutic agents in IBD. Considering that human KRT10, FGG, and TLR4 genes play an important role in the infection by these bacteria, based on the ceRNA networks, lncRNA XIST and NEAT1 suggested to play an important role in regulating the function of hsa-miR-6875-5p, hsa-miR-1908-5p, hsa-miR-186-5p, hsa-miR-6763-5p, hsa-miR-4436a, and hsa-miR-520a-5p, which may regulate the activity of genes involved in the occurrence of staphylococcus aureus and pertussis bacterial infections. The production of circRNAs in NM_001039703 and NM_006267 positions affect miR-186-5p, miR-4436a, miR-520a-5p, miR-186-5p, miR-6763-5p, and miR-520a-5p that act on the functions of KRT10, FGG, and TLR4 genes. The more experimental studies can determine the precise roles of these ncRNAs and genes in the process of causing inflammatory bowel disease. We also predicted the most effective cores for siRNAs to affect staphylococcus aureus (ClfB, IsdA, sdrC, sdrD, and SasG) and pertussis (PtxE, PagP, PtxD, PtxC, PtxA, and PtxB) bacterial genes that interacted with human KRT10, FGG, and TLR4 genes. These genes are also known in other bacteria. However, the aforesaid putative genes and ncRNAs in IBD should therefore be confirmed by additional research and experiments with larger datasets.

## Supplementary Information

Supplementary Information 1.

Supplementary Information 2.

Supplementary Information 3.

Supplementary Information 4.

Supplementary Information 5.

Supplementary Information 6.

Supplementary Information 7.

Supplementary Information 8.

## Data Availability

The datasets (GSE126124, GSE87473, GSE75214, and GSE95095) used during the current study are available from NCBI, GEO (https://www.ncbi.nlm.nih.gov/geo/). However, other data analyzed in the article can present by the corresponding author on reasonable request.

## Abbreviations

IBD: Inflammatory bowel disease
CD: Crohn's disease
UC: Ulcerative colitis
DEGs: Differential expression genes
GEO: Gene Expression Omnibus
ncRNA: Non-coding RNA
miRNA: MicroRNA
siRNA: Small interfering RNA
lncRNA: Long non-coding RNA
circRNA: Circular RNA
ceRNA: Competitive endogenous RNA
UTR: Untranslated region
MRSA: Methicillin-resistant staphylococcus aureus
NAFLD: Non-alcoholic fatty liver disease

## References

[1] Schreiber S, Rosenstiel P, Albrecht M, Hampe J, Krawczak M (2005). Genetics of Crohn disease, an archetypal inflammatory barrier disease. Nat. Rev. Genet..

[2] Szigethy E, McLafferty L, Goyal A (2010). Inflammatory bowel disease. Child Adolesc. Psychiatr. Clin. N. Am..

[3] Papaconstantinou I, Zeglinas C, Gazouli M, Nastos K, Yiallourou A, Lykoudis P (2014). Effect of infliximab on the healing of intestinal anastomosis: An experimental study in rats. Int. J. Surg..

[4] Norouzinia M, Chaleshi V, Alizadeh AHM, Zali MR (2017). Biomarkers in inflammatory bowel diseases: Insight into diagnosis, prognosis and treatment. Gastroenterol. Hepatol. Bed Bench..

[5] Kaplan GG, Ng SC (2017). Understanding and preventing the global increase of inflammatory bowel disease. Gastroenterology.

[6] Odes HS, Fich A, Reif S, Halak A, Lavy A, Keter D (2001). Effects of current cigarette smoking on clinical course of Crohn’s disease and ulcerative colitis. Dig. Dis. Sci..

[7] Higuchi LM, Khalili H, Chan AT, Richter JM, Bousvaros A, Fuchs CS (2012). A prospective study of cigarette smoking and the risk of inflammatory bowel disease in women. Am. J. Gastroenterol..

[8] Kostic AD, Ramink XJ, GD, (2014). The microbiome in inflammatory bowel disease: current status and the future ahead. Gastroenterology.

[9] Halfvarson J, Brislawn CJ, Lamendella R, Vázquez-Baeza Y, Walters WA, Bramer LM, D'Amato M, Bonfiglio F (2017). Dynamics of the human gut microbiome in inflammatory bowel disease. Nat. Microbiol..

[10] Dickinson RJ, Varian SA, Axon ATR, Cooke EM (1980). Increased incidence of faecal coliforms with in vitro adhesive and invasive properties in patients with ulcerative colitis. Gut.

[11] Hu W, Fang T, Chen X (2022). Identification of differentially expressed genes and miRNAs for ulcerative colitis using bioinformatics analysis. Front. Genet..

[12] Chen ZA, Sun YF, Wang QX, Ma HH, Ma ZZ, Yang CJ (2021). Integrated analysis of multiple microarray studies to identify novel gene signatures in ulcerative colitis. Front. Genet..

[13] Cheng F, Li Q, Wang J, Zeng F, Wang K, Zhang Y (2020). Identification of differential intestinal mucosa transcriptomic biomarkers for ulcerative colitis by bioinformatics analysis. Dis. Mark..

[14] Xiao X, Mao X, Chen D, Yu B, He J, Yan H (2022). miRNAs Can Affect Intestinal Epithelial Barrier in Inflammatory Bowel Disease. Front. Immunol..

[15] Lin L, Zhou G, Chen P, Wang Y, Han J, Chen M (2020). Which long noncoding RNAs and circular RNAs contribute to inflammatory bowel disease?. Cell Death Dis..

[16] Yarani R, Mirza AH, Kaur S, Pociot F (2018). The emerging role of lncrnas in inflammatory bowel disease. Exp. Mol. Med..

[17] Paraskevi A, Theodoropoulos G, Papaconstantinou I, Mantzaris G, Nikiteas N, Gazouli M (2012). Circulating MicroRNA in inflammatory bowel disease. J. Crohn’s Colitis..

[18] Chen W-X, Ren L-H, Shi R-H (2014). Implication of miRNAs for inflammatory bowel disease treatment: Systematic review. World J. Gastrointest. Pathophysiol..

[19] Feng X, Wang H, Ye S, Guan J, Tan W, Cheng S (2012). Up-Regulation of microRNA-126 May Contribute to Pathogenesis of Ulcerative Colitis via Regulating NF-kappaB Inhibitor IκBα. PLoS ONE.

[20] Huang YK, Yu JC (2015). Circulating microRNAs and long non-coding RNAs in gastric cancer diagnosis: An update and review. World J. Gastroenterol..

[21] Yu L, Gong X, Sun L, Zhou Q, Lu B, Zhu L (2016). The circular RNA Cdr1as act as an oncogene in hepatocellular carcinoma through targeting miR-7 expression. PLoS ONE.

[22] Yu Y, Zhang M, Liu J, Xu B, Yang J, Wang N (2018). Long non-coding RNA PVT1 promotes cell proliferation and migration by silencing ANGPTL4 expression in cholangiocarcinoma. Mol. Ther. – Nucl. Acids.

[23] Dang HX, White NM, Rozycki EB, Felsheim BM, Watson MA, Govindan R (2020). Long non-coding RNA LCAL62 / LINC00261 is associated with lung adenocarcinoma prognosis. Heliyon.

[24] Chen Q, Zhu C, Jin Y, Si X, Jiao W, He W (2020). Plasma long non-coding RNA RP11-438N5.3 as a novel biomarker for non-small cell lung cancer. Cancer Manag. Res..

[25] Ni H, Li W, Zhuge Y, Xu S, Wang Y, Chen Y (2019). Inhibition of circHIPK3 prevents angiotensin II-induced cardiac fibrosis by sponging miR-29b-3p. Int. J. Cardiol..

[26] Palmer NP, Silvester JA, Lee JJ, Beam AL, Fried I, Valtchinov VI (2019). Concordance between gene expression in peripheral whole blood and colonic tissue in children with inflammatory bowel disease. PLoS ONE.

[27] Li K, Strauss R, Ouahed J, Chan D, Telesco SE, Shouval DS (2018). Molecular comparison of adult and pediatric ulcerative colitis indicates broad similarity of molecular pathways in disease tissue. J. Pediatr. Gastroenterol. Nutr..

[28] Vancamelbeke M, Vanuytsel T, Farré R (2019). Europe PMC funders group genetic and transcriptomic basis of intestinal epithelial barrier dysfunction in inflammatory bowel disease. Inflam. Bowel Dis..

[29] Zhao, X. Differential Gene Expression between involved and uninvolved sites of Crohn’s disease: Insights into a Distinctive Pathogenesis Profile. Guangzhou (2019). https://www.ncbi.nlm.nih.gov/geo/query/acc.cgi?acc=GSE95095.

[30] Szklarczyk D, Gable AL, Nastou KC, Lyon D, Kirsch R, Pyysalo S (2021). The STRING database in 2021: Customizable protein-protein networks, and functional characterization of user-uploaded gene/measurement sets. Nucl. Acids Res..

[31] Assenov Y, Ramírez F, Schelhorn SESE, Lengauer T, Albrecht M (2008). Computing topological parameters of biological networks. Bioinformatics.

[32] Harris MA, Clark J, Ireland A, Lomax J, Ashburner M, Foulger R (2004). The Gene Oncology (GO) database and informatics resource. Nucl. Acids Res..

[33] Bindea G, Mlecnik B, Hackl H, Charoentong P, Tosolini M, Kirilovsky A (2009). ClueGO: A Cytoscape plug-in to decipher functionally grouped gene ontology and pathway annotation networks. Bioinformatics.

[34] Gautier L, Cope L, Bolstad BM, Irizarry RA (2004). Affy—Analysis of Affymetrix GeneChip data at the probe level. Bioinformatics.

[35] Leek JT, Johnson WE, Parker HS, Jaffe AE, Storey JD (2012). The SVA package for removing batch effects and other unwanted variation in high-throughput experiments. Bioinformatics.

[36] Ritchie ME, Phipson B, Wu D, Hu Y, Law CW, Shi W (2015). Limma powers differential expression analyses for RNA-sequencing and microarray studies. Nucl. Acids Res..

[37] Costello CM, Mah N, Häsler R, Rosenstiel P, Waetzig GH, Hahn A (2005). Dissection of the inflammatory bowel disease transcriptome using genome-wide cDNA microarrays. PLoS Med..

[38] Ray K (2017). Steatohepatitis: PARP inhibition protective against alcoholic steatohepatitis and NASH. Nat. Rev. Gastroenterol. Hepatol..

[39] Cheng C, Hua J, Tan J, Qian W, Zhang L, Hou X (2019). Identification of differentially expressed genes, associated functional terms pathways, and candidate diagnostic biomarkers in inflammatory bowel diseases by bioinformatics analysis. Exp. Ther. Med..

[40] Cecchini MJ, Hosein K, Howlett CJ, Joseph M, Mura M (2018). Comprehensive gene expression profiling identifies distinct and overlapping transcriptional profiles in non-specific interstitial pneumonia and idiopathic pulmonary fibrosis. Respir. Res..

[41] Baumgart DC, Carding SR (2007). Series Gastroenterology 1 Infl ammatory bowel disease : cause and immunobiology. Lancet.

[42] Albertson DG, Pinkel D (2003). Genomic microarrays in human genetic disease and cancer. Hum. Mol. Genet..

[43] Salas A, Hernandez-Rocha C, Duijvestein M, Faubion W, McGovern D, Vermeire S, Vetrano S, Vande CN (2020). JAK-STAT pathway targeting for the treatment of inflammatory bowel disease. Nat. Rev. Gastroenterol. Hepatol..

[44] Li M, Li P, Tang R, Lu H (2022). Resveratrol and its derivates improve inflammatory bowel disease by targeting gut microbiota and inflammatory signaling pathways. Food. Sci. Hum. Wellness..

[45] Sairenji T, Collins KL, Evans DV (2017). An update on inflammatory bowel disease. Prim Care - Clin Off Pract..

[46] Elguezabal N, Chamorro S, Molina E, Garrido JM, Izeta A, Rodrigo L, Juste RA (2012). Lactase persistence, NOD2 status and Mycobacterium avium subsp paratuberculosis infection associations to Inflammatory Bowel Disease. Gut. Pathog..

[47] Stockman JA (2006). Culture of Mycobacterium avium subspecies paratuberculosis from the blood of patients with Crohn’s disease. Yearb Pediatr..

[48] Sartor RB (2005). Does Mycobacterium avium subspecies paratuberculosis cause Crohn’s disease?. Gut.

[49] Mishina D, Katsel P, Brown ST, Gilberts ECAM, Greenstein RJ (1996). On the etiology of Crohn disease. Proc. Natl. Acad. Sci. USA.

[50] Sartor B (2009). Microbial-host interactions in inflammatory bowel diseases and experimental colitis. Nestle Nutr. Work Ser. Pediatr. Progr..

[51] Balfour SR (2007). Bacteria in Crohn’s disease: Mechanisms of inflammation and therapeutic implications. J. Clin. Gastroenterol..

[52] Wertheim HFL, Melles DC, Vos MC, Van Leeuwen W, Van Belkum A, Verbrugh HA (2005). The role of nasal carriage in Staphylococcus aureus infections. Lancet Infect Dis..

[53] Lindberg E, Adlerberth I, Hesselmar B, Saalman R, Strannegård IL, Åberg N (2004). High rate of transfer of staphylococcus aureus from parental skin to infant gut flora. J. Clin. Microbiol..

[54] Nguyen GC, Patel H, Chong RY (2010). Increased prevalence of and associated mortality with methicillin-resistant staphylococcus aureus among hospitalized IBD patients. Am. J. Gastroenterol..

[55] Leung W, Malhi G, Willey BM, McGeer AJ, Borgundvaag B, Thanabalan R (2012). Prevalence and predictors of MRSA, ESBL, and VRE colonization in the ambulatory IBD population. J. Crohn’s Colitis..

[56] Moriggi M, Pastorelli L, Torretta E, Tontini GE, Capitanio D, Bogetto SF (2017). Contribution of extracellular matrix and signal mechanotransduction to epithelial cell damage in inflammatory bowel Disease patients: A proteomic study. Proteomics.

[57] Owens DW, Wilson NJ, Hill AJM, Rugg EL, Porter RM, Hutcheson AM (2004). Human keratin 8 mutations that disturb filament assembly observed in inflammatory bowel disease patients. J. Cell Sci..

[58] Appay V, Sauce D (2008). Immune activation and inflammation in HIV-1 infection: causes and consequences. J. Pathol..

[59] Walsh EJ, O’Brien LM, Liang X, Hook M, Foster TJ (2004). Clumping factor B, a fibrinogen-binding MSCRAMM (microbial surface components recognizing adhesive matrix molecules) adhesin of Staphylococcus aureus, also binds to the tail region of type I cytokeratin 10. J. Biol. Chem..

[60] Schaffer AC, Solinga RM, Cocchiaro J, Portoles M, Kiser KB, Risley A (2006). Immunization with Staphylococcus aureus clumping factor B, a major determinant in nasal carriage, reduces nasal colonization in a murine model. Infect. Immun..

[61] Lipsky BA, Pecoraro RE, Chen MS, Koepsell TD (1987). Factors affecting staphylococcal colonization among NIDDM outpatients. Diabetes Care.

[62] Corrigan RM, Miajlovic H, Foster TJ (2009). Surface proteins that promote adherence of Staphylococcus aureus to human desquamated nasal epithelial cells. BMC Microbiol..

[63] Roche FM, Meehan M, Foster TJ (2003). The Staphylococcus aureus surface protein SasG and its homologues promote bacterial adherence to human desquamated nasal epithelial cells. Microbiology.

[64] Marr N, Hajjar AM, Shah NR, Novikov A, Yam CS, Caroff M (2010). Substitution of the bordetella pertussis lipid a phosphate groups with glucosamine is required for robust nf-κb activation and release of proinflammatory cytokines in cells expressing human but not murine toll-like receptor 4-MD-2-CD14. Infect. Immun..

[65] Visintin, A., Iliev, D. B., Monks, B. G., Halmen, K. A., Golenbock, D. T. Md-2. *Immunobiology*. **211**, 437–447 (2006). 10.1016/j.imbio.2006.05.010 16920483

[66] Buchheister S, Buettner M, Basic M, Noack A, Breves G, Buchen B (2017). CD14 Plays a Protective Role in Experimental Inflammatory Bowel Disease by Enhancing Intestinal Barrier Function. Am. J. Pathol..

[67] Li G, Lin J, Zhang C, Gao H, Lu H, Gao X (2021). Microbiota metabolite butyrate constrains neutrophil functions and ameliorates mucosal inflammation in inflammatory bowel disease. Gut. Microbes..

[68] Mangmool S, Kurose H (2011). Gi/o protein-dependent and -independent actions of pertussis toxin (ptx). Toxins (Basel)..

[69] Bishop RE, Gibbons HS, Guina T, Trent MS, Miller SI, Raetz CRH (2000). Transfer of palmitate from phospholipids to lipid A in outer membranes of Gram-negative bacteria. EMBO J..

[70] Cheng Q, Wang L (2020). LncRNA XIST serves as a ceRNA to regulate the expression of ASF1A, BRWD1M, and PFKFB2 in kidney transplant acute kidney injury via sponging hsa-miR-212–3p and hsa-miR-122–5p. Cell Cycle.

[71] Li S, Liu X, Li H, Pan H, Acharya A, Deng Y (2018). Integrated analysis of long noncoding RNA-associated competing endogenous RNA network in periodontitis. J. Periodontal. Res..

[72] Jing Z, Guo S, Zhang P, Liang Z (2020). LncRNA-Associated ceRNA Network Reveals Novel Potential Biomarkers of Laryngeal Squamous Cell Carcinoma. Technol. Cancer Res. Treat..

[73] He T, Lf L, Gj D, Wt W, Sc C, Ml K (2012). Pro-opiomelanocortin gene delivery suppresses the growth of established Lewis lung carcinoma through a melanocortin-1 receptor-independent pathway. J. Gene Med..

[74] Ye YL, Yin J, Hu T, Zhang LP, Wu LY, Pang Z (2019). Increased circulating circular RNA-103516 is a novel biomarker for inflammatory bowel disease in adult patients. World J. Gastroenterol..

[75] Padua D, Mahurkar-Joshi S, Law IKM, Polytarchou C, Vu JP, Pisegna JR (2016). A long noncoding RNA signature for ulcerative colitis identifies IFNG-AS1 as an enhancer of inflammation. Am. J. Physiol. – Gastrointest. Liver Physiol..

[76] Ma M, Pei Y, Wang X, Feng J, Zhang Y, Gao MQ (2019). LncRNA XIST mediates bovine mammary epithelial cell inflammatory response via NF-κB/NLRP3 inflammasome pathway. Cell Prolif..

[77] Zhou Z, Ni H, Li Y, Jiang B (2021). LncRNA XIST promotes inflammation by downregulating GRα expression in the adenoids of children with OSAHS. Exp. Ther. Med..

[78] Liu R, Tang A, Wang X, Chen X, Zhao L, Xiao Z (2018). Inhibition of lncRNA NEAT1 suppresses the inflammatory response in IBD by modulating the intestinal epithelial barrier and by exosome-mediated polarization of macrophages. Int. J. Mol. Med..

[79] Pan S, Liu R, Wu X, Ma K, Luo W, Nie K (2021). LncRNA NEAT1 mediates intestinal inflammation by regulating TNFRSF1B. Ann. Transl. Med..

[80] Wang K, Zhang Z, Liu K, Yang X, Zou H, Zhou J (2018). Neat1-miRNA204-5p-PI3K-AKT axis as a potential mechanism for photodynamic therapy treated colitis in mice. Photodiagnosis Photodyn. Ther..

[81] Bu F, Wang A, Zhu Y, You H, Zhang Y, Meng X (2020). LncRNA NEAT1: Shedding light on mechanisms and opportunities in liver diseases. Liver Int..

[82] Yanagihara K, Tashiro M, Fukuda Y, Ohno H, Higashiyama Y, Miyazaki Y (2006). Effects of short interfering RNA against methicillin-resistant Staphylococcus aureus coagulase in vitro and in vivo. J. Antimicrob. Chemother..

[83] Zhang Y, Cristofaro P, Silbermann R, Pusch O, Boden D, Konkin T (2006). Engineering mucosal RNA interference in vivo. Mol Ther..

[84] Xiao B, Laroui H, Viennois E, Ayyadurai S, Charania MA, Zhang Y (2014). Nanoparticles with surface antibody against CD98 and carrying CD98 small interfering RNA reduce colitis in mice. Gastroenterology.

