# Supplementary figures and images for: Cross talk between bacterial and human gene networks enriched using ncRNAs in IBD disease
Source: Sci Rep. 2023 May 11;13:7704. doi: 10.1038/s41598-023-34780-x (PMC10175251; doi:10.1038/s41598-023-34780-x)

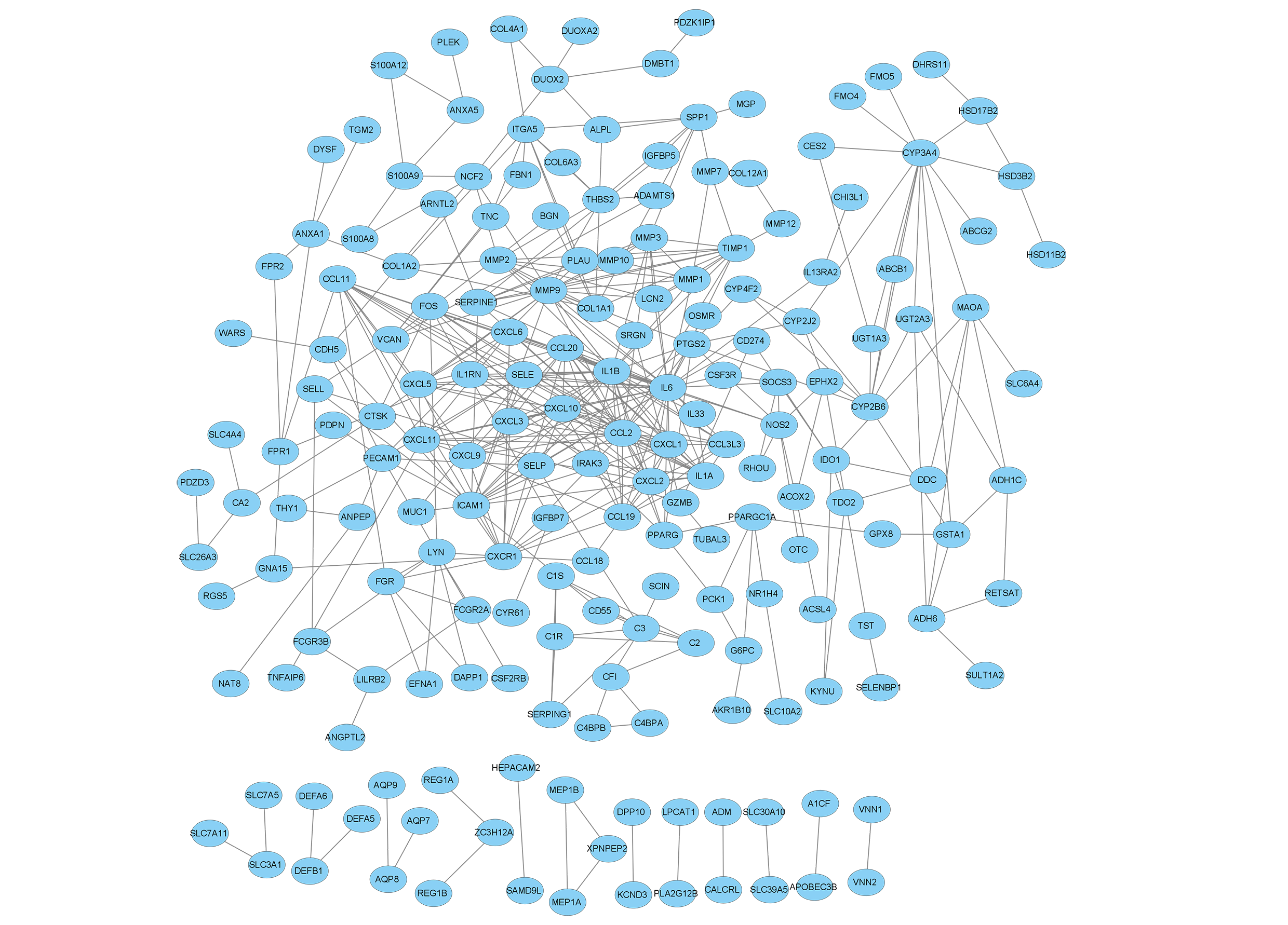

Supplement: Supplementary file 2 — Supplementary Information 2. [file 41598_2023_34780_MOESM2_ESM.tif]

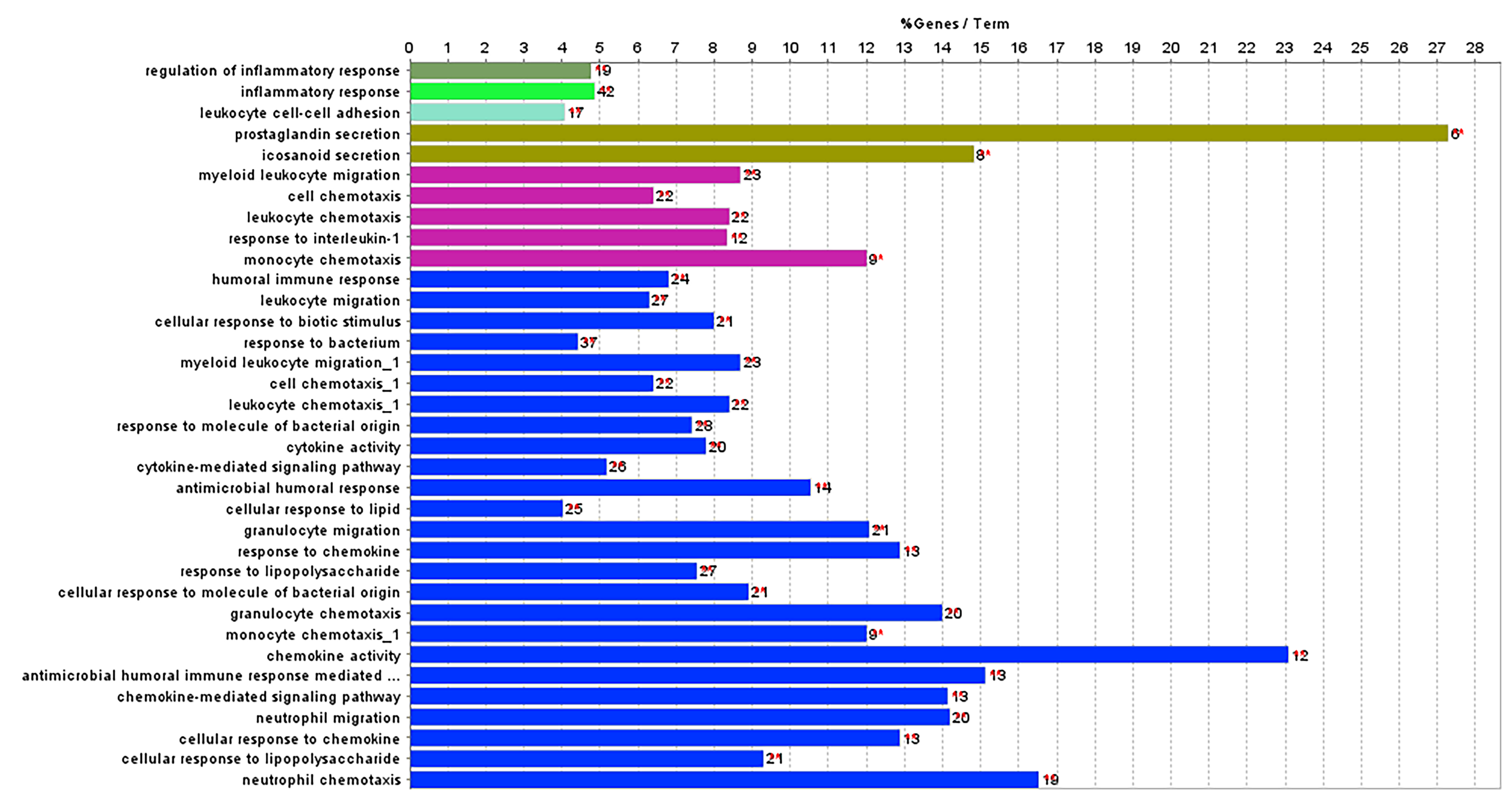

Supplement: Supplementary file 3 — Supplementary Information 3. [file 41598_2023_34780_MOESM3_ESM.tif]

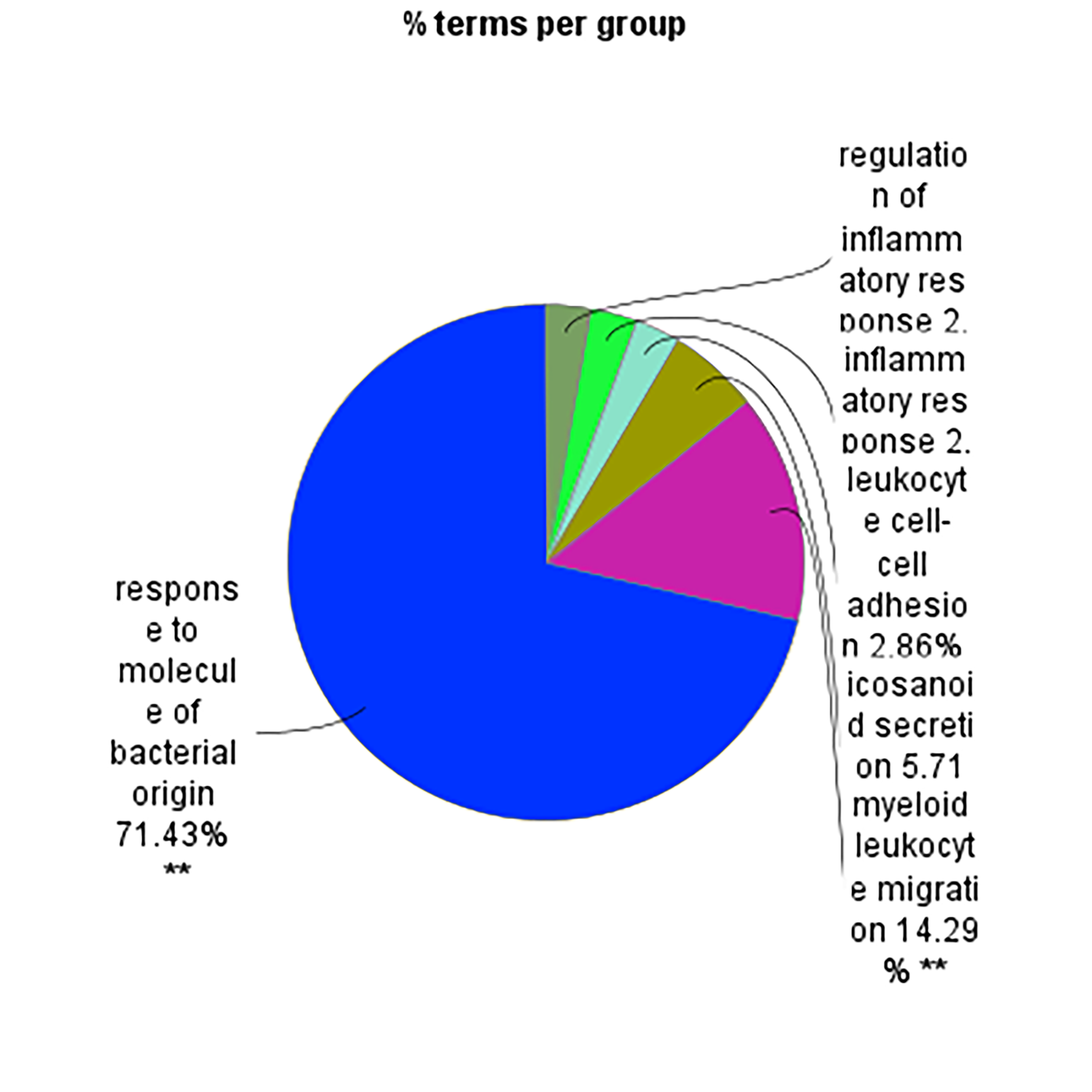

Supplement: Supplementary file 4 — Supplementary Information 4. [file 41598_2023_34780_MOESM4_ESM.tif]

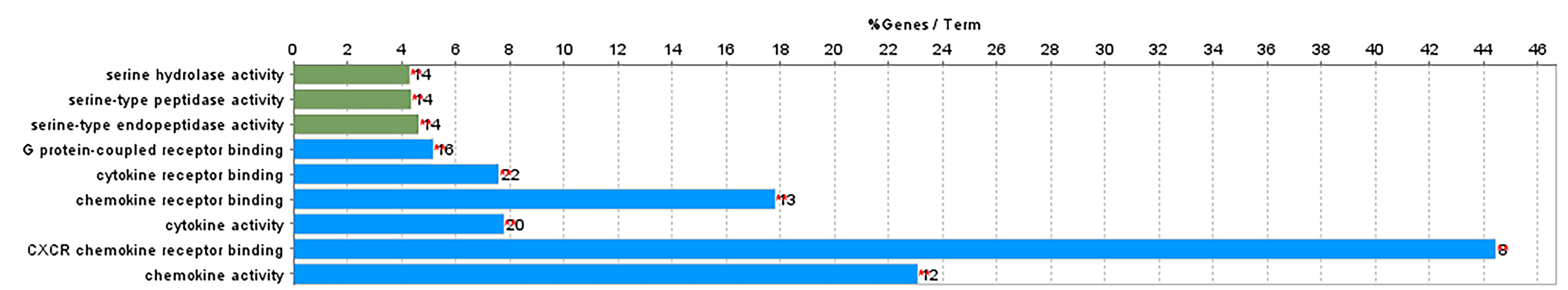

Supplement: Supplementary file 5 — Supplementary Information 5. [file 41598_2023_34780_MOESM5_ESM.tif]

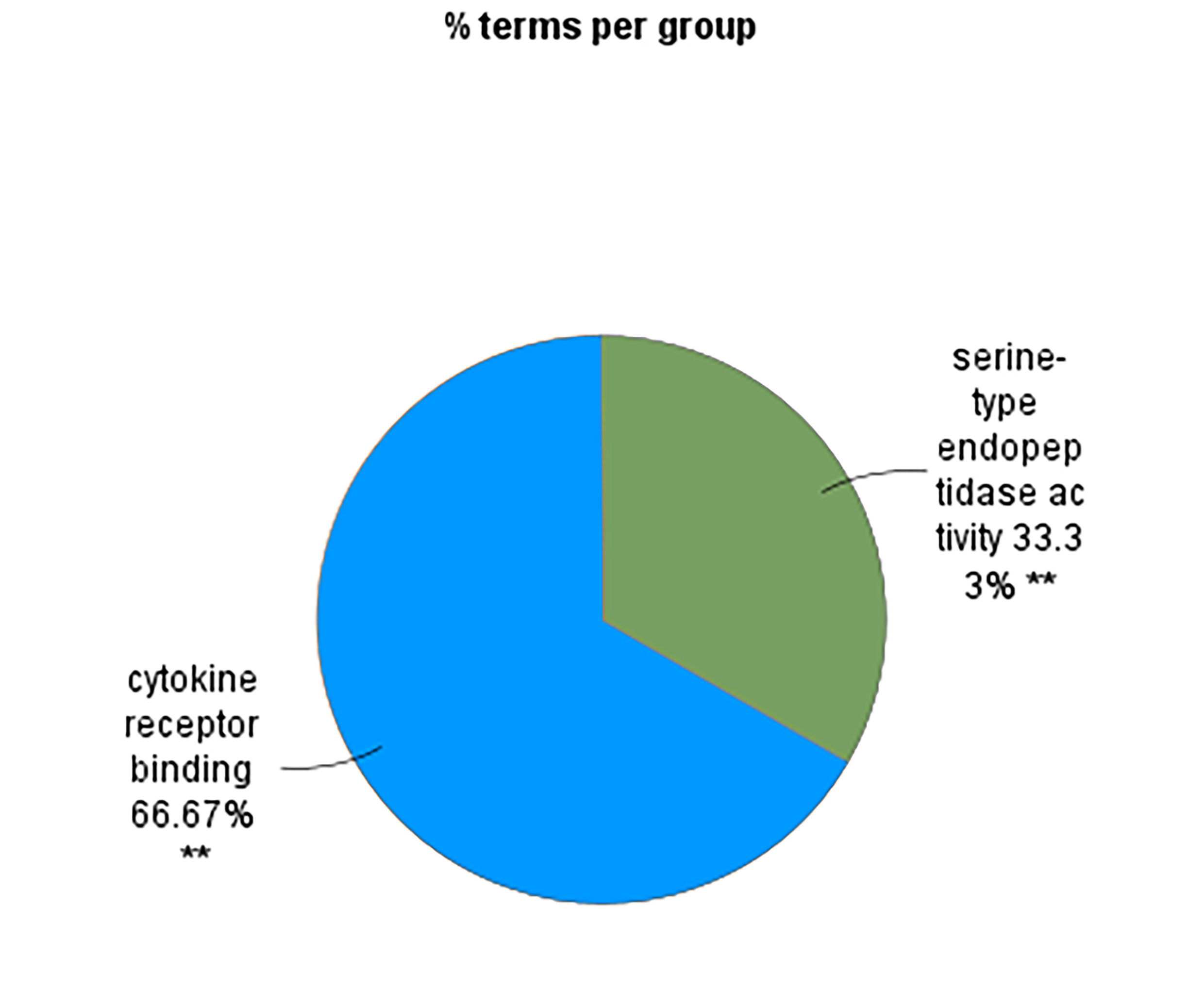

Supplement: Supplementary file 6 — Supplementary Information 6. [file 41598_2023_34780_MOESM6_ESM.tif]

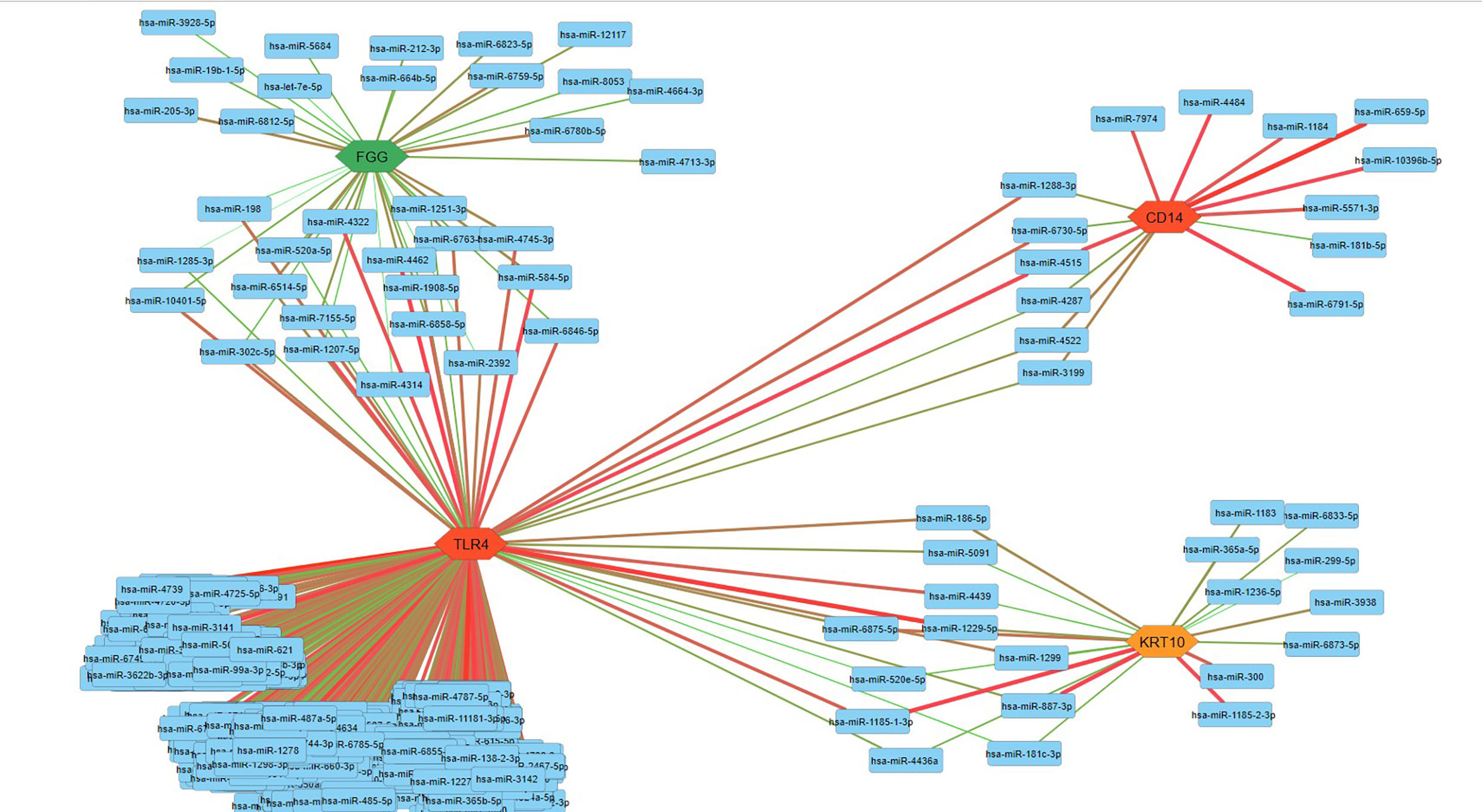

Supplement: Supplementary file 7 — Supplementary Information 7. [file 41598_2023_34780_MOESM7_ESM.tif]

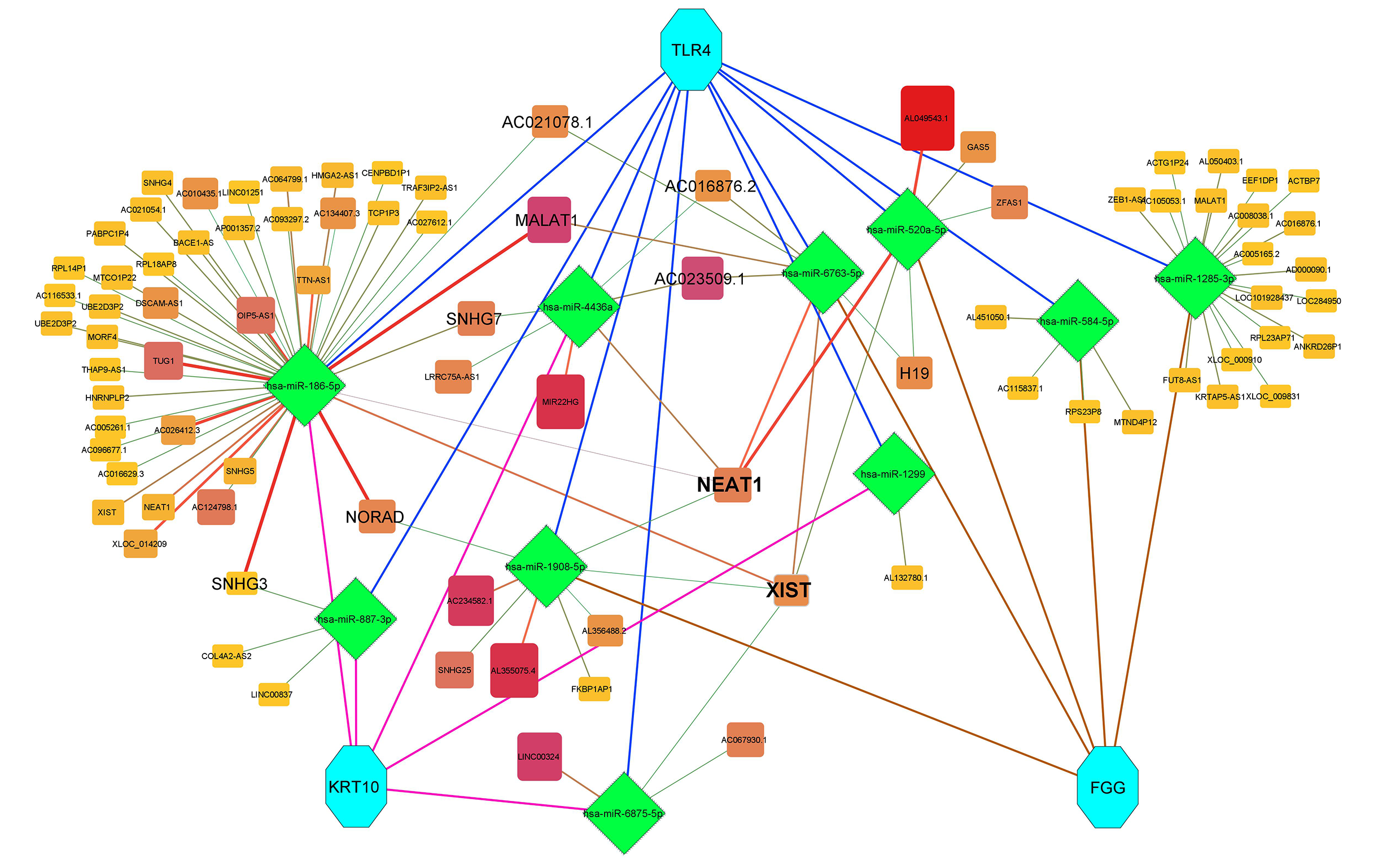

Supplement: Supplementary file 8 — Supplementary Information 8. [file 41598_2023_34780_MOESM8_ESM.tif]
